# Injectable biodegradable microcarriers for iPSC expansion and cardiomyocyte differentiation

**DOI:** 10.1002/advs.202404355

**Published:** 2024-06-20

**Authors:** Annalisa Bettini, Patrizia Camelliti, Daniel J. Stuckey, Richard M. Day

**Affiliations:** ^1^ Centre for Advanced Biomedical Imaging, Division of Medicine University College London London WC1E 6DD UK; ^2^ Centre for Precision Healthcare, Division of Medicine University College London London WC1E 6JF UK; ^3^ School of Biosciences and Medicine University of Surrey Guildford Surrey GU2 7XH UK

**Keywords:** cardiac regeneration, cardiomyocyte maturation, injectable biomaterials, microcarriers, stem cells

## Abstract

Cell therapy is a potential novel treatment for cardiac regeneration and numerous studies have attempted to transplant cells to regenerate the myocardium lost during myocardial infarction. To date, only minimal improvements to cardiac function have been reported. This is likely to be the result of low cell retention and survival following transplantation. This study aimed to improve the delivery and engraftment of viable cells by using an injectable microcarrier that provides an implantable, biodegradable substrate for attachment and growth of cardiomyocytes derived from induced pluripotent stem cells (iPSC). We describe the fabrication and characterisation of Thermally Induced Phase Separation (TIPS) microcarriers and their surface modification to enable iPSC‐derived cardiomyocyte attachment in xeno‐free conditions is described. The selected formulation resulted in iPSC attachment, expansion, and retention of pluripotent phenotype. Differentiation of iPSC into cardiomyocytes on the microcarriers is investigated in comparison with culture on 2D tissue culture plastic surfaces. Microcarrier culture is shown to support culture of a mature cardiomyocyte phenotype, be compatible with injectable delivery, and reduce anoikis. The findings from this study demonstrate that TIPS microcarriers provide a supporting matrix for culturing iPSC and iPSC‐derived cardiomyocytes in vitro and are suitable as an injectable cell‐substrate for cardiac regeneration.

## Graphical abstract

A novel type of implantable biodegradable polymer microcarrier fabricated using thermally induced phase separation is designed to facilitate the attachment and differentiation of iPSC‐derived cardiomyocytes. The injectable microcarriers address existing challenges associated with use of iPSC‐derived cardiomyocyte cell‐based therapy, including the impact of cell anoikis during bioprocessing, clinical compatibility, and the production of physiologically relevant cardiomyocytes.

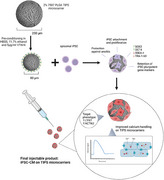

## 1 Introduction

Myocardial infarction leading to chronic heart failure (CHF) is a major healthcare burden. Although treatments exist to manage the symptoms there is currently no cure available. There is, therefore, an increasing interest in the use of regenerative therapies to provide a curative treatment for CHF.

Cell therapies have the potential to repair damaged organs including the heart. Many studies have attempted to transplant cells to regenerate myocardium lost as a result of tissue infarction. Existing strategies generally consist of delivering anchorage dependent cells, such as mesenchymal stem cells, in an unanchored, unnatural, suspended state.^[^ ^1^, ^2^ ^]^ Furthermore, the majority of adult stem cells are unable to generate an indefinite number of pure cardiomyocytes (CM) necessary to achieve effective tissue regeneration.^[^ ^3^, ^4^, ^5^, ^6^ ^]^ Investigators are now exploring other potential cell sources, such as induced pluripotent stem cells (iPSC). iPSC can propagate indefinitely and can be guided into specific lineages, providing sufficient quantities of cells that could replace damaged or diseased tissue, including the human myocardium.^[^ ^7^ ^]^ However, to date, the preclinical benefits of cardiac cell therapy have only produced modest improvements in clinical trials, irrespective of the cell type used.^[^ ^3^, ^8^ ^]^

Effective cell therapy for treatment of CHF requires targeted delivery of cells to the required location followed by their long term survival and functional integration with the myocardium. The therapeutic effect is dependent on survival of transplanted cells.^[^ ^9^, ^10^, ^11^, ^12^ ^]^ Therefore, viable cells must be retained in the myocardium to enable them to engraft and ultimately contribute to muscle contractility and/or send paracrine signals to host cells to stimulate regeneration. Intravenous, intracoronary, and trans‐endocardial delivery of therapies consisting of cells in suspension are generally performed by minimally invasive catheter injections.^[^ ^12^ ^]^ However, cell retention via these delivery routes can be poor due to washout effects and cell death.^[^ ^12^ ^]^ A materials‐based approach offers a potential solution to enhance cell retention by providing a scaffold for cell attachment and the creation of a tissue engineered construct.^[^ ^13^, ^14^ ^]^

To date, materials‐based approaches for myocardial regeneration have typically utilized surgically implanted patches or injectable hydrogels.^[^ ^15^, ^16^, ^17^, ^18^, ^19^, ^20^, ^21^, ^22^ ^]^ Injectable hydrogels have been generated from a range of synthetic and natural materials including, alginate, PEG, chitosan and PLGA.^[^ ^16^, ^23^, ^24^ ^]^ Advantages of hydrogels include simple mixing with cells and biologics just prior to injection and minimally invasive delivery of a liquid via small gauge needles before crosslinking in situ. Gelation and degradation times can be modified by the choice of material and cross‐linking mechanism.^[^ ^16^, ^23^, ^24^ ^]^ Injectable hydrogels are clinically desirable as they avoid the need for surgical implantation but cell‐cell interaction and structural organisation is often missing, resulting in lower cell retention compared with implanted 3D constructs.^[^ ^13^, ^25^ ^]^

Cardiac patches and engineered heart tissues (EHTs) are often comprised of cells attached to a scaffold, which encourages cell survival and facilitates cell‐cell contact resulting in structured cellularised constructs.^[^ ^26^ ^]^ Structured materials have been shown to improve iPSC‐CM based tissue maturation, electrical coupling and longer cell retention in vivo.^[^ ^13^, ^19^, ^20^ ^]^ However, to deliver cardiac patches to the heart, epicardial attachment is normally performed via direct surgical access to the heart through a thoracotomy.^[^ ^19^, ^25^, ^27^, ^28^, ^29^ ^]^ The invasive open‐chest surgery increases the risk of intervention‐related morbidities and limits the clinical application.^[^ ^12^ ^]^

Therefore, an injectable material that can also support cell attachment and cell‐cell interaction would provide benefits from both hydrogel and cardiac patch approaches by enabling minimally invasive delivery of a tissue engineered construct into the myocardium to support local integration and better cell survival.

Microcarriers are an effective technology for cellular bioprocessing applications. They often consist of polymerized materials with a diameter of 100–400 µm and a surface that has been optimized for cell attachment.^[^ ^30^ ^]^ Microcarriers have generally been developed for two types of cellular applications: (i) in vitro cell expansion and production of biologics, or (ii) in vivo cell delivery for regenerative medicine. For in vivo delivery, microcarriers have the benefit of being administered via minimally invasive injections for localized delivery to the target site.^[^ ^31^, ^32^ ^]^ Additionally, microcarriers can be designed and optimized for use with specific types of anchorage dependent cells.^[^ ^31^ ^]^ The physical properties of the microcarrier, such as porosity, surface chemistry, and degradability can be customized by appropriate selection of the starting polymer, crosslinking parameters, and post‐synthetic modifications. Microcarriers have been extensively investigated for the large‐scale expansion of iPSC to produce quantities of cells needed for clinical use.^[^ ^33^ ^]^ The production of iPSC‐derived cardiomyocytes (iPSC‐CM) on microcarriers has also been described.^[^ ^34^, ^35^, ^36^ ^]^ However, the production of iPSC‐CM under xeno‐free conditions using injectable microcarrier delivery systems has not previously been reported.

Thermally Induced Phase Separation (TIPS) microcarriers offer all of the required attributes outlined above for cell expansion and in vivo delivery, making them an attractive candidate for cardiac regenerative applications. They provide a structured material for cell attachment, which is also injectable and compatible for minimally invasive delivery. TIPS microcarriers have previously been shown to be an excellent substrate for the cellular attachment of skeletal myoblasts, mesenchymal stem cells and several other cell types.^[^ ^37^, ^38^, ^39^, ^40^, ^41^, ^42^ ^]^ Additionally, the microcarriers have been shown to integrate and retain in vivo after implantation. The fabrication process for TIPS microcarriers is flexible and it can be scaled‐up for clinical use. However, to date it is not known whether TIPS microcarriers are suitable for culture of iPSC‐CM and delivery to cardiac tissues.

Here, we have developed an injectable cell‐substrate consisting of highly porous TIPS microcarriers that enable the attachment and expansion of iPSC in xeno free‐conditions. Microcarrier attachment was shown to be protective against anoikis, supportive of mature cardiomyocyte phenotype, and compatible for injectable delivery. This work demonstrates that TIPS microcarriers offer a supporting matrix for culturing iPSC and iPSC‐CM in vitro and may provide an injectable substrate for cardiac regeneration.

## 2 Results

### 2.1 Selection and optimization of TIPS microcarrier formulation for iPSC attachment

**Figure 1:**
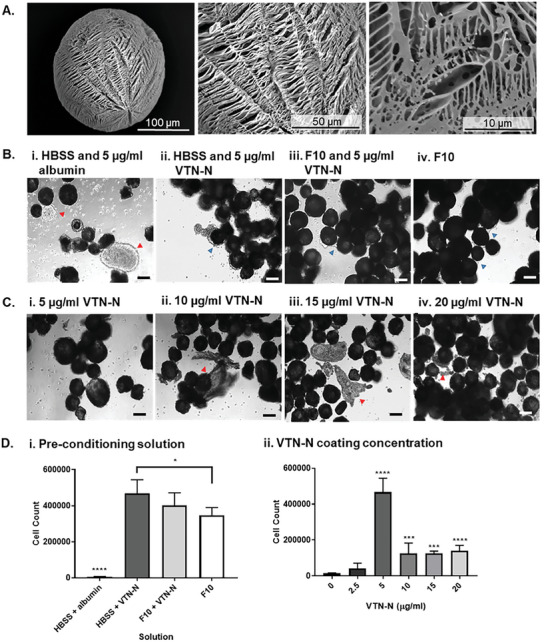
Optimization of TIPS microcarriers for iPSC attachment. A) Ultrastructural features of the 2% 7507 TIPS microcarriers were assessed using SEM. B) and C) Conditions for pre‐conditioning TIPS microcarrier to facilitate iPSC attachment. 5 × 10^5^ P6 iPSC were seeded on 20 mg of microcarriers pre‐conditioned with either HBSS and 5 µg/ml albumin (negative control), HBSS and 0–20 µg/ml VTN‐N, complete F10 media and 5 µg/ml VTN‐N and complete F10 media (positive control). iPSC were attached under static dynamic conditions (30 seconds gentle agitation at 30 RPM every hour) for 24 hours. D) After 24 hours, iPSC attachment to the TIPS microcarriers was quantified using a NucleoCounter NC‐200 automated cell counter. Red arrows show cellular clumping and uneven attachment of cells to the microcarriers. Blue arrows show cell attachment to the microcarriers, with formation of cellular connections between microcarriers. Scale bars represent 100 µm. Data are presented as mean ±SD. The significance of the data was calculated by two‐way ANOVA, Tukey's post‐hoc correction. (n = 6, *P ≤ 0.05, ***P ≤ 0.001, ****P ≤ 0.0001).

To identify TIPS microcarrier pre‐conditioning conditions that enabled iPSC attachment, a range of compositions were investigated. A 2 (w/v) % 7507 PLGA polymer composition was chosen for the formulation of the TIPS microcarriers (Figure S1). SEM demonstrated that 2% 7507 PLGA polymer processed by TIPS produced microcarriers with rough and highly porous surface topographies (**Figure** **1**A). The pores ranged in shape and size and some pores appeared interconnected.

TIPS microcarriers did not initially mix with aqueous media due to PLGA being hydrophobic. Therefore the microcarriers underwent a pre‐conditioning process to render them hydrophilic to enable cell‐material interaction.^[^ ^43^ ^]^ Pre‐conditioning was achieved by mixing the microcarriers in a solution containing ethanol, as previously described.^[^ ^44^ ^]^ The pre‐conditioning of the microcarriers was initially investigated using a solution composed of 10% ethanol and complete F10 media. To increase cell attachment to the microcarriers, adsorption of extracellular matrix proteins to the surface of the microcarriers was investigated (Figure S1). iPSC cell attachment to the microcarriers was visually assessed using light microscopy and quantified at 24 hours (Figure 1B–D). Supplementing complete F10 media pre‐conditioning solution with VTN‐N increased the number of iPSC attached to the microcarriers but this was not significant compared with F10 media alone (Figure 1Di). Switching the pre‐conditioning solution to HBSS, ethanol and VTN‐N resulted in the highest iPSC attachment (Figure 1Di). The use of HBSS and albumin as a pre‐conditioning solution resulted in a low number of iPSC attaching to the microcarriers (Figure 1B).

The effect of different concentrations of VTN‐N in the pre‐conditioning solution on iPSC attachment was further investigated. Microcarriers were pre‐conditioned with a range of 0–20 µg/ml VTN‐N in HBSS pre‐conditioning solutions. The highest number iPSC attachment was achieved with 5 µg/ml VTN‐N (Figure 1C and Dii). At concentrations higher than 5 µg/ml VTN‐N, the number of cells attached to the microcarriers decreased (Figure 1C), with attached cells clumping on the surface rather than evenly spreading on the surface of the microcarriers (Figure 1Dii).

### 2.2 Cell culture on TIPS microcarriers enables cell proliferation and confers protection against anoikis

**Figure 2:**
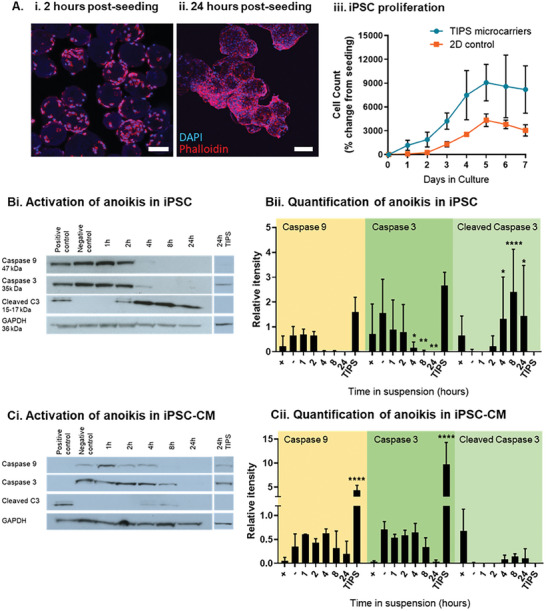
iPSC attachment to the TIPS microcarriers leads to cell proliferation and protection against anoikis. A) iPSC proliferate on the surface of TIPS microcarriers: fluorescence microscopy images of iPSC attached to TIPS microcarriers stained for nuclei (DAPI blue) and cytoskeleton (phalloidin red), after (i) 2 hours, and (ii) 24 hours of seeding. Scale bars represent 100 µm. (iii) Proliferation of iPSC seeded on TIPS microcarriers and 2D tissue culture plastic surfaces, over 7 days in culture. Attachment of cells to the TIPS microcarriers for 24 hours appears to reduce anoikis, as indicated by the expression downstream markers of apoptosis, inactive caspases 3 and 9, and active cleaved caspase 3. Western blot image of B) iPSC and C) iPSC‐CM in suspension up to 24 hours or attached to TIPS microcarriers for 24 hours. (ii) Protein expression was quantified relative to GAPDH. Hours (h), cleaved caspase 3 (cleaved C3), 24 hour attachment to the TIPS microcarriers (TIPS). Positive controls for the onset of cell death were treated with 100 µM etoposide for 4 hours. Data are presented as mean ±SD. The significance of the data was calculated two‐way ANOVA with Dunnett's post‐hoc correction. (n = 4, *P ≤ 0.05, **P ≤ 0.01, ****P ≤ 0.0001, statistics to matched negative control).

TIPS microcarriers pre‐conditioned in 5 µg/ml VTN‐N HBSS solution were selected for iPSC attachment. The approach used to select the microcarrier formulation is summarised in Figure S2. Confocal microscopy revealed iPSC adhesion to the microcarriers (**Figure** **2**A). Light microscopy analysis showed that after 1 hour of seeding iPSC were tethered to the surface of microcarriers and exhibited a rounded morphology, suggesting non‐complete attachment. After 2 hours from seeding, the cells appeared more attached, with cellular spreading over the surface of the microcarriers. At 24 hours after seeding, iPSC appeared to cover the whole surface of the microcarriers, as well as cellular bridging occurring between adjacent microcarriers (Figure 2Aii). Cells attached to the microcarriers proliferated during 7 days of culture (Figure 2Aiii).

Attachment of iPSC and iPSC‐CM to TIPS microcarriers for 24 hours protected cells from anoikis (Figure 2B and C). 24 hours post‐seeding, cells attached to TIPS microcarriers retained high expression of inactive caspase 3 and caspase 9 (Figure 2B and C), which are precursors to downstream markers of apoptosis activation via anoikis.^[^ ^45^, ^46^ ^]^ Concurrently, expression of active cleaved caspase 3, an indicator of cell death, was not detected. Positive controls treated with 100 µM etoposide for 4 hours (known to activate apoptosis via G1 cell cycle arrest^[^ ^47^ ^]^), as well as maintaining cells cultured in suspension for up to 24 hours resulted in inactive caspase 3 and caspase 9 levels being decreased while activated forms of caspase‐3 increased (Figure 2B and C). Similarly, flow cytometric analysis demonstrated that cells attached to TIPS microcarriers for 24 hours retained high expression of viability marker Cytocalcein violet 450, while reducing the expression of apoptosis and necrosis markers, Phosphatidylserine and DNA Nuclear green DCS1, compared to samples cultured in suspension (Figure S11).

### 2.3 iPSC culture on TIPS microcarriers retains pluripotent phenotype

**Figure 3:**
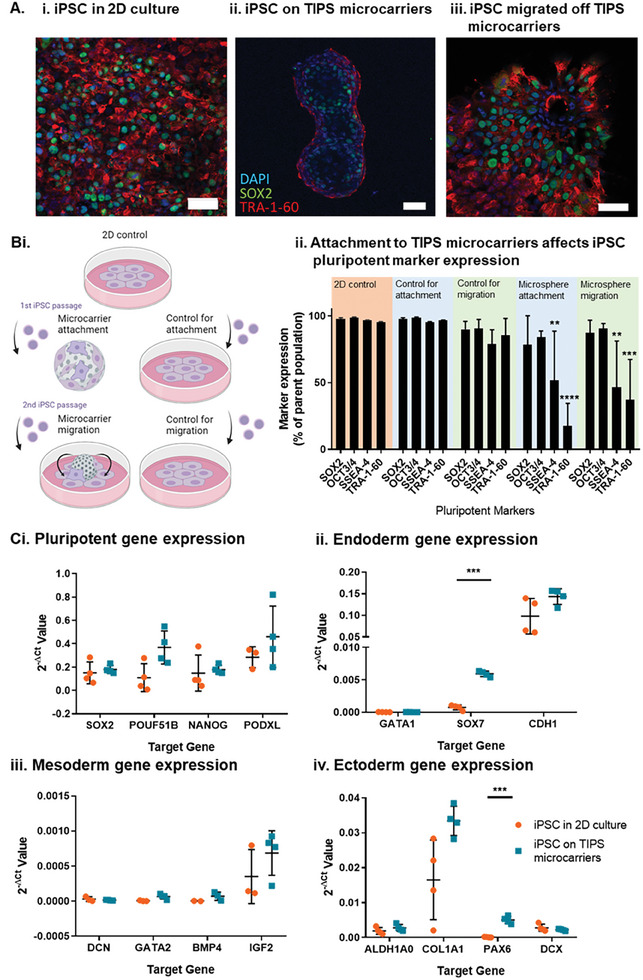
Characterization of iPSC pluripotent phenotype by A) confocal microscopy, B) flow cytometry and C) qRT‐PCR. Retention of iPSC pluripotency markers, SOX2 (green), TRA‐1‐60 (red) and DAPI (blue), in (Ai) 2D control tissue culture plastic conditions, (ii) attached to TIPS microcarriers for 24 hours and (iii) migrated for 72 hours, off TIPS microcarriers and onto 2D control conditions. B) Flow cytometric assessment of iPSC phenotype in different culture conditions and matched passaging controls. Attachment and migration controls were investigated, matching the passaging of cells and length of culture conditions, with re‐attachment onto 2D control conditions. Attachment samples consisted of iPSC passaged from 2D tissue culture conditions and seeded onto TIPS microcarriers or VTN‐N coated plates for 24 hours (control). Migration samples, consisted of iPSC passaged first from 2D tissue culture conditions and seeded on TIPS microcarriers or VTN‐N coated plates (control), and either migrated for 72 hours off TIPS microcarriers onto VTN‐N coated plates or (control) passaged a second time from the VTN‐N coated plate onto a fresh VTN‐N coated plates for 72 hours to match passage number and culture conditions. C) Trilineage differentiation marker expression in iPSC attached to TIPS microcarriers. Changes in (i) pluripotent, (ii) endoderm, (iii) mesoderm, and (iv) ectoderm gene expression in iPSC attached to TIPS microcarriers compared with iPSC grown on 2D control tissue culture surface. Gene expression was calculated relative to the expression of the house keeping gene glyceraldehyde‐3‐phosphate dehydrogenase (GAPDH). Scale bar represents 50 µm. Data are presented as mean ±SD. The significance of the data was calculated by (B) Two‐way ANOVA, Dunnett's post‐hoc correction and (C) unpaired t‐test with Holm‐Sidak's post hoc analysis. (n = 3‐4, **P ≤ 0.01, ***P ≤ 0.001 ****P ≤ 0.0001, statistics to matched 2D control).

To investigate whether attachment of iPSC to the TIPS microcarriers affected iPSC phenotype, pluripotency marker expression was investigated. Confocal microscopy confirmed the retention of iPSC pluripotency markers after 24 hours of attachment to the microcarriers via the expression of SOX2 nuclear marker and TRA‐1‐60 cell surface marker (**Figure** **3**Aii and Figure S12). Positive staining for TRA‐1‐60 was visible on the outline of the cellularised TIPS microspheres. The distribution of TRA1‐60 expression conforms with the expression patterns reported for iPSC staining in literature, including staining of episomal iPSC on Synthemax II coated dissolvable microcarriers.^[^ ^48^, ^49^, ^50^ ^]^ After 24 hours on TIPS microcarriers, iPSC were migrated off TIPS microcarriers onto VTN‐N coated tissue culture plastic for 72 hours static incubation. This approach was used to investigate the effect of microcarrier interaction on iPSC pluripotency under conditions that simulated pre‐ and post‐ cellularisation of the microcarriers. Migrated iPSC stained positively for SOX2 and TRA‐1‐60 (Figure 3Aiii) and the distribution of staining was similar to control iPSC cultured on 2D tissue culture plastic surfaces (Figure 3Ai).

Flow cytometry analysis of the expression of pluripotent markers SOX2, OCT4, TRA‐1‐60 and SSEA‐4 in cells detached from TIPS microcarriers (gating parameters in Figure S3) indicated attachment of iPSC to TIPS microcarriers for 24 hours affected the expression of extracellular pluripotent markers, with the expression of SSEA‐4 and TRA‐1‐60 reduced after culture on TIPS microcarriers (Figure 3Bii). Following TIPS microcarrier attachment for 24 hours, migration onto tissue culture plastic surfaces for 72 hours also resulted in reduced expression of extracellular markers SSEA‐4 and TRA‐1‐60, compared with iPSC cultured on control 2D tissue culture plastic surfaces. The changes in pluripotent marker expression were not dependent on the cellular passaging associated with the cell attachment to the microcarriers. Attachment and migration controls were investigated, matching the passaging of cells and length of culture conditions, with re‐attachment onto 2D tissue culture plastic surfaces. The attachment control was comparable to the 2D tissue culture plastic surfaces, with pluripotent marker expression levels retained (>95%). The migration control, matched for 2 passages, trended towards reduced pluripotent marker expression (>75%). However, the reduction was not statistically significant compared to control cells cultured on the 2D tissue culture surfaces (Figure 2Bii).

To investigate whether interaction of iPSC with TIPS microcarriers was associated with spontaneous cell differentiation, a qPCR panel was designed to probe for early markers of trilineage differentiation (Figure 3C). Attachment of iPSC to TIPS microcarriers did not affect the genetic expression of pluripotent markers. Pluripotent markers expression was comparable to iPSC cultured on 2D tissue culture plastic surfaces. iPSC attachment to TIPS microcarriers increased the expression of endoderm gene *SOX7* and ectoderm gene *PAX6* relative to cells cultured on 2D tissue culture plastic surfaces, however, the expression levels were trivial.

Retention of pluripotent cell phenotype was investigated by the ability of iPSC to differentiate. iPSC were migrated under static culture conditions from the microcarriers onto VTN‐N coated tissue culture plastic (Figure S5). Migrated iPSC were expanded (Figure S5A) and further differentiated into iPSC‐CM (Figure S5B and C). The differentiated cells began to spontaneously beat from day 8 of differentiation (Figure S5D) and continued to do so for up to 40 days (Figure S5E and F). The ability of migrated iPSC to form beating cardiac bundles aligns with the results from Figure 3, suggesting that iPSC pluripotency and ability to differentiate was retained after attachment and culture on TIPS microcarriers.

### 2.4 Attachment of terminally differentiated and enriched iPSC‐CM to TIPS microcarriers

**Figure 4:**
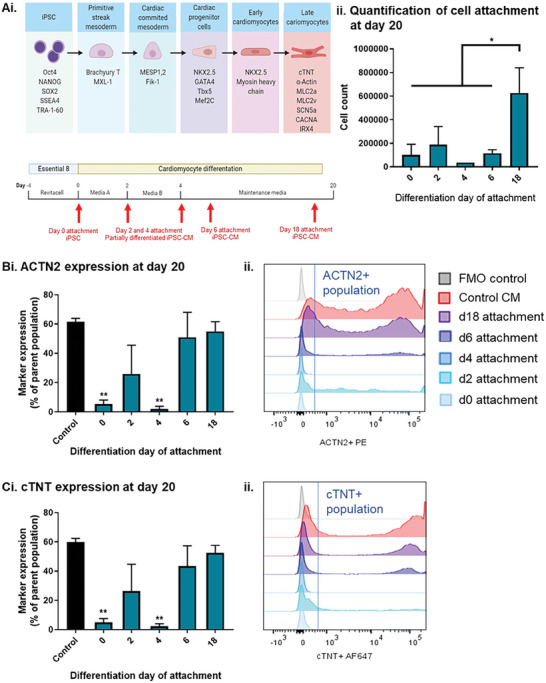
Assessing cardiac differentiation of iPSC on TIPS microcarriers. Ai) Schematic of CM differentiation strategy and corresponding cell phenotypes for attachment to TIPS microcarriers. Red arrows denote the time points at which the partly differentiated and differentiated CM were attached to the TIPS microcarriers. iPSC at day 0, partially differentiated iPSC‐CM, at day 2 and 4, and iPSC‐CM, at day 6 and 18 of differentiation were attached to TIPS microcarriers. After attachment, cells were terminally differentiated into cardiomyocytes up to day 20. (ii) Quantification of iPSC‐CM attached to TIPS microcarriers at day 20. Flow cytometric characterisation of the expression of cardiac markers B) ACTN2 and C) cTNT at day 20, in iPSC‐CM attached to TIPS microcarriers. The expression of the markers was (i) quantified and (ii) representative histograms of quantified samples plotted against tissue culture and FMO controls. Data are presented as mean ±SD. The significance of the data was calculated by One‐way ANOVA with Dunnett's post hoc analysis. (n = 4, *P ≤ 0.05 and **P ≤ 0.01, statistics to matched control CM).

Differentiation of iPSC to cardiomyocytes while attached to TIPS microcarriers was investigated using a commercially available kit (Thermo Fisher) based on the protocol developed by Burridge et al.^[^ ^51^ ^]^ Differentiation parameters were optimized for the episomal iPSC line, as described in Figure S6. Time‐points for the attachment of cells to TIPS microcarriers at different stages of differentiation (iPSC at day 0, partially differentiated iPSC‐CM, at day 2 and 4, and iPSC‐CM, at day 6 and 18 of differentiation) is denoted by red arrows in **Figure** **4**Ai. After attachment, the differentiation protocol continued up to day 20. At day 20, the number of cells attached to the microcarriers was quantified (Figure 4Aii). Cells attached to microcarriers on days 0, 2, 4 and 6 of differentiation resulted in cell counts of <2 × 10^5^ cells on day 20. However, iPSC‐CM attached on day 18 resulted in cells counts of >5 × 10^5^ cells. At day 20, the phenotype of cells attached to the microcarriers was assessed for cardiac phenotype by flow cytometry analysis (Figure 4B and C). The expression of cardiac markers, cardiac troponin (cTNT) and sarcomeric alpha actinin (ACTN2) was compared with a 2D tissue culture control (gating strategy illustrated in Figure S4). iPSC differentiated from day 0 and day 4 on TIPS microcarriers expressed lower levels of cTNT and ACTN2 compared with the control iPSC‐CM differentiated from day 2 primitive streak mesodermal cells on TIPS microcarriers. Expression levels were not different to the 2D tissue culture plastic control group. Attachment of iPSC‐CM from day 6 and 18 post differentiation to TIPS microcarriers yielded cells with a similar expression of cardiac markers to the control group. These data suggest that iPSC, or partially differentiated iPSC, could not be differentiated into iPSC‐CM onto TIPS microcarriers using the current approach. However, attachment of pre‐differentiated iPSC‐CM to the TIPS microcarriers enabled the retention of a cardiac phenotype, which was comparable to the phenotype of cardiac cells differentiated on tissue culture plastic.

**Figure 5:**
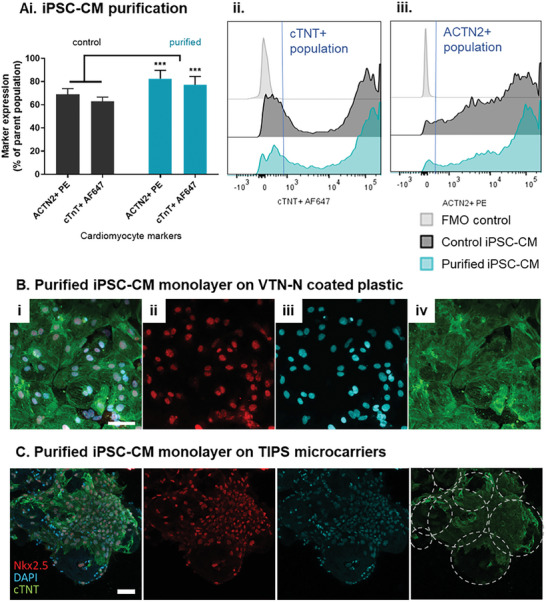
Magnetic purification of iPSC‐CM. A) Flow cytometric characterisation of the expression of cardiac markers cTNT and ACTN2 in iPSC‐CM purified on day 18 of differentiation. After purification, iPSC‐CM were seeded on VTN‐N coated plastic dishes for 2D control and TIPS microcarriers for 7 days to produce monolayers. Confocal micrographs of purified iPSC‐CM monolayer on VTN‐N coated plastic dish B) and on TIPS microcarriers C) 7 days post‐seeding. Visualisation of (ii) NKx2.5 in red, (iii) DAPI in blue, (iv) cTNT in green and (i) composite images. Microcarriers outlined by dotted lines. Scale bar represents 50 µm. Data are presented as mean ±SD. The significance of the data was calculated by two‐way ANOVA with Sidak's post hoc analysis. (n = 4, ***P ≤ 0.001, statistics to matched control CM).

Attachment of iPSC‐CM to TIPS microcarriers on day 18 resulted in the highest cell number of attached cells and expression of cardiac markers. Therefore this composition was used for further experiments. Magnetic‐activated cell sorting was performed to enrich the iPSC‐CM population. Flow cytometry analysis indicated that the depletion of non‐CMs was effective (**Figure** **5**A and Figure S13), with purified cells expressing higher ACTN2 and cTNT cardiac markers compared with the non‐purified cell population. The enriched iPSC‐CM were re‐plated to produce confluent monolayers on VTN‐N coated dishes and cultured for 7 days (Figure 5B). Enriched iPSC‐CM attached to TIPS microcarriers spontaneously contracted from day 2 onwards, which was observed as spontaneous movement of microcarriers in suspension. The enriched iPSC‐CM on TIPS microcarriers also expressed high levels of cTNT and NKx2.5 (Figure 5C).

### 2.5 iPSC‐CM cellularized TIPS microcarriers are injectable and show improved calcium cycling

**Figure 6:**
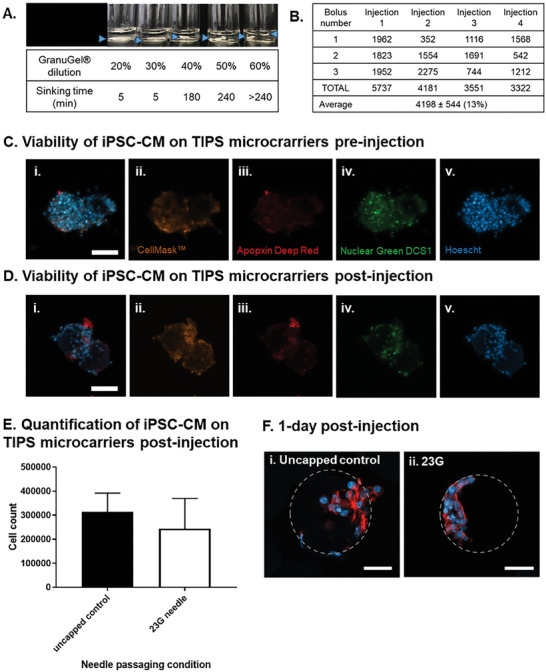
Cellularised TIPS microcarriers are injectable. A) Selection of the GranuGel dilution for delivery of TIPS microcarriers in suspension. Blue arrows indicate the location of the sinking TIPS microcarriers suspension in the tube. 1 × 10^6^ enriched Day 18 iPSC‐CM were seeded on 20 mg of microcarriers and incubated under static dynamic conditions for 24 hours. The media was replaced and the cells were left to recover for another 72 hours. 4 days after cell seeding, the sample was resuspended in 600 µL of 60% GranuGel and injected through either a 23G needle capped or an uncapped syringe into a 24 well low‐bind plate and the samples were immediately analysed or cultured in EB 2% media for 6 days. B) Quantification of the number of TIPS microcarriers delivered in an injection. Each injection consisted of 3 boluses of 50 µL for the total delivery volume of 150 µL. C,D) The viability of iPSC‐CM on TIPS microcarriers was assessed pre‐ and post‐injection (i) Composite image acquired from over imposing (ii) CellMask™ orange plasma membrane stain, (iii) Apopxin deep red, (iv) necrosis marker Nuclear green DCS1 and (v) viability marker Hoescht. E) The mean number of purified iPSC‐CM remaining attached to TIPS microcarriers following delivery through a 23G needle was not different compared with the number of cells remaining attached to microcarriers following delivery through an uncapped syringe. The number of cells in the sample was quantified using a Chemometec automated cell counter. F) 1 day after injection samples were fixed and stained for nuclear (DAPI blue) and cytoskeleton (phalloidin red) markers. Cellularised microcarriers injected through (i) Control = uncapped syringe, (ii) 23G = 23G needle capped syringe. Scale bars represent 50 µm. Microcarriers are outlined in grey. Data are presented as mean ±SD. The significance of the data was calculated by unpaired two‐tailed student's *t*‐test. (n = 5‐6, p = NS).

The TIPS microcarriers loaded with iPSC‐CM are intended to be an injectable therapeutic product that is compatible with minimally invasive delivery into the myocardium. To deliver the cell laden microcarriers in a uniform suspension that avoids sedimentation in the syringe, a viscosity modifier consisting of GranuGel was used (**Figure** **6**A). Diluting GranuGel to 60% (v/v) was selected to retain the microcarriers in suspension while still allowing the composition to pass through a 23G needle. Dilutions of 70% (v/v) were more viscous and difficult to deliver through the needle and therefore not selected. Previous studies have demonstrated it is feasible to deliver 3 × 50 µL bolus injections into the myocardium of rats.^[^ ^52^ ^]^ The number of TIPS microcarriers loaded in each bolus was quantified using a semi‐automated Morphologi G3 particle size and particle shape image analyzer to estimate the number of TIPS microcarriers deliverable in vivo (Figure 6B).

Retention of cell viability following delivery through a syringe and needle is a key attribute if the iPSC CM‐laden TIPS microcarriers are going to be investigated in vivo. To assess this, day 18 enriched iPSC‐CM on TIPS microcarriers were suspended in GranuGel and injected through a 23G needle capped syringe. The injected microcarriers were analysed immediately or cultured for a further 6 days. iPSC‐CM attachment to the microcarriers post‐injection was confirmed by confocal microscopy (Figure 6C, D and F). The majority of cells attached to TIPS microcarriers remained viable (Figure 6D). A low number of cells exhibited an apoptotic and necrotic phenotype, as indicated by positive staining for apopxin red and necrotic marker nuclear green DCS1. The expression pattern in injected cells on TIPS microcarriers (Figure 6D) was comparable to iPSC‐CM on TIPS microcarriers pre‐injection (Figure 6C). The absence of a noticeable change to the level of staining indicates that there was no discernible change in viability of cells post‐injection. Cell attachment to TIPS microcarriers following delivery through the 23G needle was similar to the control, consisting of TIPS microcarriers delivered through an uncapped syringe group (Figure 6E), and the cells retained their spontaneous beating characteristics up to 7 days post‐injection (see Video 1).

**Figure 7:**
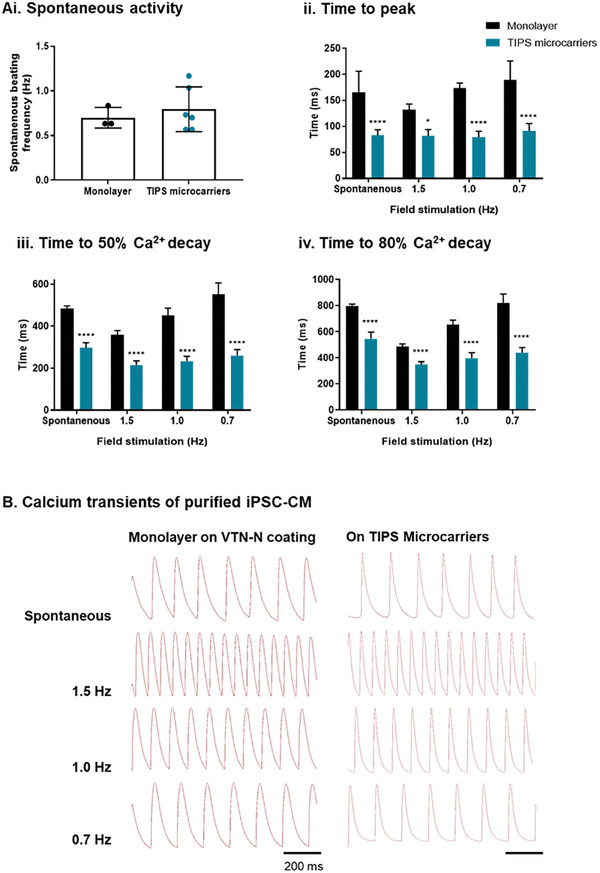
Assessment of iPSC‐CM calcium handling. A) Quantification of (i) spontaneous activity and (ii‐iv) calcium handing of purified day 18 iPSC‐CM seeded as confluent monolayers on VTN‐N coated dishes and TIPS microcarriers, cultured for 7 days post‐purification. The spontaneous transient was compared with electrical field‐stimulated iPSC‐CM at 0.7, 1.0 and 1.5 Hz. Calcium imaging quantified measurements of time to (ii) transient peak, (iii) 50% Ca^2+^ decay and (iv) 80% Ca^2+^ decay. Statistics to matched iPSC‐CM on TIPS microcarriers. B) Representative calcium transient traces. Scale bar represents 200 ms. Data are presented as mean ±SD. The significance of the data was calculated by one‐way ANOVA with Holm Sidak's multiple comparison test. (n = 3‐11, *P ≤ 0.05, ****P ≤ 0.0001).

The spontaneous activity observed in the enriched iPSC‐CM monolayers and iPSC‐CM on TIPS microcarriers was assessed by quantification of spontaneous beating frequencies. Calcium handling was also investigated using optical mapping. The calcium transients of spontaneous and electrical field stimulated iPSC‐CM at 0.7 Hz, 1.0 Hz and 1.5 Hz were compared (**Figure** **7**A). Calcium transient analysis revealed that the purified iPSC‐CM plated in control monolayer conditions and on TIPS microcarriers showed physiological calcium cycling. Representative optical calcium transients in Figure 7B also show that iPSC‐CM on TIPS microcarriers respond to a range of external electrical stimuli (0.7‐1.5 Hz), including the physiological relevant frequency of 1.0 Hz. Spontaneously beating iPSC‐CM on VTN‐N pre‐conditioned TIPS microcarriers had shorter time to peak, time to 50% Ca^2+^ decay, and time to 80% Ca^2+^ decay compared with iPSC‐CM cultured on VTN‐N coated tissue culture dishes. Electrical field stimulation of enriched iPSC‐CM attached to TIPS microcarriers showed Ca^2+^ cycling was affected, with shorter time to peak, quicker time to 50% Ca^2+^ decay, and quicker time to 80% Ca^2+^ decay, compared with electrically stimulated iPSC‐CM on VTN‐N coated tissue culture substrates.

## 3 Discussion

Poor cell survival, retention and engraftment are challenges that impede the intended benefit of regenerative cardiac cell therapy. To improve the viability of transplanted cells, an injectable material was developed that supports cell attachment, maintains cell viability, and is capable of withstanding minimally invasive delivery.

The microcarriers were optimised for iPSC‐derived cardiac cell attachment using a pre‐conditioning solution that resulted in them becoming hydrophilic, similar to that described elsewhere for other material cell‐interactions.^[^ ^39^, ^44^ ^]^ Pre‐conditioning was achieved by incubating the microcarriers in a xeno‐free pre‐conditioning solution selected for stem cell culture consisting of HBSS, ethanol and, VTN‐N. Compatibility of the microcarriers with iPSC culture was assessed by evaluation of cellular adhesion, growth kinetics and retention of pluripotent phenotype. Confocal microscopy confirmed iPSC attachment to the microcarriers after 2 hours of seeding. After 24 hours, iPSC covered the surface of the microcarriers and cellular bridging was visible between iPSC on adjacent microcarriers. Proliferation of iPSC attached to the microcarriers was observed over 7 days, with the cell number increasing linearly, followed by exponential growth that subsequently plateaued. These growth kinetics are comparable to published literature.^[^ ^53^ ^]^ These data indicate that microcarriers were not cytotoxic and compatible with iPSC culture.

Retention of a relevant cellular phenotype after attachment to the microcarriers is essential for achieving the intended therapeutic outcome. Assessment of several molecular markers can identify the pluripotent status of iPSC, such as expression of cell surface proteins TRA‐1‐60 and SSEA4, and the transcription factors OCT4, SOX2 and NANOG.^[^ ^54^ ^]^ Biocompatibility of the microcarriers with attached iPSC was indicated by retention of the pluripotent markers SOX2 and TRA‐1‐60. However, the expression of SSEA‐4 and TRA‐1‐60 were reduced after culture on TIPS microcarriers and subsequent detachment, indicating attachment of iPSC to TIPS microcarriers affected the expression of these extracellular pluripotent markers. The reduction in pluripotent marker expression did not appear to be dependent on passaging following cell detachment, with control samples matched for passaging retaining pluripotent marker. In contrast to the reduced expression of extracellular pluripotent cell markers, pluripotent nuclear marker expression was retained.

To further investigate whether interaction of iPSC with TIPS microcarriers was associated with spontaneous cell differentiation, a qPCR panel was designed to probe for early markers of trilineage differentiation. The markers chosen were based on the studies by Granata et al.^[^ ^55^ ^]^ and D'Antonio et al.^[^ ^56^ ^]^

The expression of pluripotent and differentiation markers was compared to cells cultured on 2D tissue culture plastic surfaces. Attachment of iPSC to TIPS microcarriers increased the expression of endoderm gene *SOX7* and ectoderm gene *PAX6* in a statistically significant manner. However, given that expression levels were trivial, the results suggested biological insignificance. Importantly, attachment of iPSC to TIPS microcarriers did not significantly affect pluripotent gene expression. These data indicate that iPSC interaction with TIPS microcarriers retains pluripotent phenotype and does not induce spontaneous differentiation. The reduced expression of extracellular markers SSEA‐4 and TRA‐1‐60 observed by flow cytometry was likely the result of iPSC cytoskeletal interaction with the TIPS microcarriers^[^ ^57^, ^58^, ^59^ ^]^ rather than loss of pluripotency.

Having established a microcarrier composition that supports attachment and culture of pluripotent iPSC, we investigated whether iPSC can be differentiated to a cardiomyocyte phenotype while attached to the microcarriers. Attempts to directly differentiate iPSC or partly differentiated iPSC‐CM attached to the microcarriers into cells expressing cardiac troponin (cTNT) and sarcomeric alpha actinin (ACTN2) were unsuccessful. Late CM can be distinguished by the expression of cardiac markers cTNT and ACTN2, which regulate muscle contraction.

Attachment of partially differentiated iPSC‐CM led to a lower number of cells attached to the TIPS microcarriers, however the small fraction of cells attached to the TIPS microcarrier was not hindered in cardiac differentiation. This discrepancy may be due to the disruption of culture conditions during the differentiation process. Despite robust differentiation protocols and a controlled culture environment the cells are likely susceptible to mechano‐disruption, such as that occurring during passaging and re‐attachment onto a topographically different environment, including the TIPS microcarriers.

In contrast, cells initially pre‐differentiated to day 6 and day 18 before attachment to the microcarriers expressed cTNT and ACTN2 at levels comparable to cells differentiated on 2D control conditions. Attachment of day 18 pre‐differentiated iPSC‐CM to TIPS microcarriers enabled the highest level of cell attachment and this approach was used for subsequent experiments.

To deliver a more defined cell therapy, the purity of the differentiated cell population was improved to generate a greater yield of iPSC‐CM. Magnetic purification was used to enrich the proportion of cardiac iPSC‐CM at day 18 prior to TIPS microcarrier attachment. The phenotype of enriched iPSC‐CM after attachment to TIPS microcarriers (77.18% cTNT positive iPSC‐CM) was comparable with the results reported by Oh et al.^[^ ^36^, ^60^ ^]^ Oh et al. described the generation of an iPSC‐CM population that was 48.1% cTNT positive after differentiation on Cytodex I microcarriers for 10 days.^[^ ^36^, ^60^ ^]^ Further enrichment (83.1% cTNT positive CM) was achieved after purification in lactate‐supplemented medium.^[^ ^36^, ^60^ ^]^ Similarly, Laco et al. described production of >90%% cTNT positive iPSC‐CM when cultured on Cytodex I microcarriers using lactate purification. However, in the study, cardiac differentiation on Cytodex I microcarriers was observed in only one of the three cardiogenic iPSC lines tested.^[^ ^35^ ^]^ It is therefore possible that iPSC‐CM purity on the TIPS microcarriers could be further enriched using lactate purification. However, the current approach would still provide a novel approach consisting of iPSC‐CM attached to implantable TIPS microcarriers under xeno‐free conditions. Xeno‐free cardiac differentiation protocols have been reported to generate a lower percentage of iPSC‐CM yield^[^ ^61^, ^62^, ^63^ ^]^ and it would be interesting to compare other type of iPSC lines and cardiomyocyte differentiation protocols with the developed microcarrier system.

Ideally, the iPSC‐microcarrier tissue engineered construct should be compatible with minimally invasive delivery. Direct injection is a preferred method for delivering tissue engineered constructs in a minimally invasive manner rather than open surgical implantation via thoracotomy or sternotomy.^[^ ^5^, ^9^, ^64^ ^]^ Intramyocardial injections can be used to target and localize the products to specific regions of the myocardium of the LV by ultra‐sound guided transthoracic intramyocardial injections.^[^ ^52^, ^65^, ^66^ ^]^ The current study devised an injectable formulation consisting of an inert carrier vehicle (diluted GranuGel) that enabled the TIPS microcarriers to remain in suspension for over 4 hours. Previous pre‐clinical rat models have delivered between 50 µl^[^ ^67^ ^]^ to 300 µl^[^ ^68^ ^]^ of cell‐loaded material via intramyocardial injections. For example, Chow et al. delivered 3 × 50 µL depots of hydrogel containing iPSC‐CM around the myocardial infarct border zone on the LV.^[^ ^52^ ^]^ The multiple bolus system enabled the delivery of the hydrogel treatment to the top, mid and apical region of the myocardium.^[^ ^52^ ^]^ A volume of 3 × 50 µL boluses per injection was investigated in this study. The findings suggest that 4198 ± 544 microcarriers in suspension could be delivered via injection using a 23G needle, and this formulation is compatible with minimally invasive intramyocardial injections in vivo.

Delivering viable cells that survive the transplantation process is crucial for the success of cell‐based therapies. Existing approaches for delivering unattached cells often result in a large proportion of cells dying before or during transplantation, with cell viability values as low as 1–32% post‐injection.^[^ ^69^ ^]^ This may arise due to mechanical damage to the cells arising from shear forces encountered during the injection process.^[^ ^70^ ^]^ The in vitro viability experiments in the current study indicated that iPSC‐CM on TIPS microcarriers could be delivered by injection through a 23G needle capped syringe without mechanical disruption, as shown by retention of cell number and viability markers post‐injection. Furthermore, iPSC‐CM cultured in vitro following simulated delivery remained attached to the microcarriers and continue beating up to 6 days post‐injection.

Existing approaches for delivering cell‐based therapies, including those intended for cardiac regeneration are further hampered by the loss of cell viability due to anoikis, a form of apoptosis associated with detaching adherent cells from their substrate.^[^ ^9^, ^10^, ^11^, ^12^, ^71^ ^]^ A key benefit of implantable microcarriers for tissue regeneration applications would be the ability to manufacture and deliver adherent cells in a natural state, anchored to a substrate that diminishes the impact of anoikis. Western blot analysis demonstrated that cells attached to TIPS microcarriers retained high expression levels of inactive caspase 3 and caspase 9. Corresponding with this, the expression of cleaved caspase 3, an indicator of cell death, was not detected. Conversely, control samples consisting of unattached cells maintained in suspension culture resulted in inactive caspase 3 and caspase 9 levels being decreased, while activated forms of caspase‐3 increased. Flow cytometric analysis of viability, apoptosis and necrosis markers also showed that culture on TIPS microcarriers for 24 hours diminished the impact of apoptosis and necrosis compared to suspension culture. These data indicate suspension culture of cells attached to the microcarriers avoids the detrimental effects of apoptosis associated with conventional bioprocessing used for producing cell‐based therapies, which consist of producing detached adherent cells in suspension. Therefore, use of the implantable microcarriers may be beneficial for increasing the proportion of viable cells delivered that lead to improved clinical outcomes, especially since symptomatic relief in a clinical setting is associated with higher cell viability after transplantation.^[^ ^9^, ^10^, ^11^, ^12^ ^]^

The differentiated cardiac phenotype of iPSC‐CM on TIPS microcarriers was further investigated by quantifying contractile function and calcium handling. Calcium transient analysis is based on myocardial contraction and relaxation being orchestrated by calcium cycling through excitation‐contraction coupling.^[^ ^72^ ^]^ Mature cardiomyocyte contraction is coordinated by an efficiency calcium handling system. iPSC‐CM typically have slower calcium dynamics, characterised with increased time to peak and slower Ca^2+^ decay, suggestive of an immature calcium handling system.^[^ ^10^, ^73^ ^]^ Attachment of enriched iPSC‐CM to the TIPS microcarriers affected Ca^2+^ cycling, with shorter time to peak, quicker time to 50% Ca^2+^ decay and quicker time to 80% Ca^2+^ decay, compared with control monolayer cultures. The faster calcium dynamics were suggestive of improved Ca^2+^ cycling. Additionally, iPSC‐CM on TIPS microcarriers were paced at 0.7 Hz, 1.5 Hz and at a physiologically relevant frequency of 1.0 Hz. The ability of iPSC‐CM to be paced at 1 Hz suggests that engraftment of transplanted iPSC‐CM on TIPS microcarriers in vivo could couple at a physiologically relevant frequency. Additionally, the ability of iPSC‐CM on TIPS microcarriers to respond to different frequencies also shows their ability to adapt to changes in heart beating frequency. Culture of iPSC‐CM on grooved biomaterials has previously influenced maturation of the calcium handling phenotype. Rao et al. reported that iPSC‐CM cultured on a fibronectin coated micro‐grooved PDMS scaffold displayed improved sarcoplasmic reticulum Ca^2+^ handling, measured by a shortened time to peak and improved sarcoplasmic reticulum Ca^2+^ release in response to caffeine.^[^ ^74^ ^]^ The improved calcium handling was not associated with changes in gene expression but was considered to be associated with improved cellular alignment and more organised sarcomeres.^[^ ^74^ ^]^ The current study is the first to report improved calcium handling in iPSC‐CM when combined with an injectable material. Further studies are necessary to explore the use of TIPS microcarriers as a substrate to overcome the immaturity of iPSC‐CM function with a focus on quantification of Ca^2+^ extrusion, spatial organisation of calcium handling apparatus and contractile cytoskeleton, expression patterns of genes encoding proteins important in calcium regulation, and assessment of metabolic maturation.

The primary aim of this work was to develop and characterise in vitro a novel biomaterial‐based approach for culturing and delivering iPSC‐derived cardiomyocytes. While the data from the current study show the approach to be technically feasible, we recognize that further work will be necessary before the technology can be clinically translated. This includes comparison to other biomaterial delivery methods to determine whether injectable microcarriers permit higher cell retention and viability compared with hydrogels or patches. We hypothesize that the size of the microcarriers can overcome the washout effect and that administration of adherent cells in a more natural, attached state, is likely to reduce the level of cell stress during manufacturing and implantation. It will also be important to undertake in vivo investigation of iPSC‐CM maturation, structural and electrical integration with the host myocardium and contribution to cardiac function. To address this, studies should be conducted in animal models of an anatomically relevant size (most likely porcine) using clinically relevant quantities of the material and translational surrogate end‐points for evaluating the efficacy of therapy (MRI evaluation of cardiac function).

## 4 Conclusion

The current study demonstrates proof‐of‐concept for a novel injectable microcarrier system that functions as a cell‐substrate to facilitate delivery of iPSC‐CM. Notably, the microcarriers enable iPSC‐CM to retain key features of maturation and, moreover, reduce the impact of anoikis associated with conventional cell bioprocessing and delivery methods. These are important attributes that could overcome major challenges associated with cell‐based therapies for cardiac muscle and other tissues that could be used to advance the field of regenerative medicine.

## 5 Experimental Section

### Fabrication of TIPS microcarriers

Implantable microcarriers were prepared via thermally induced phase separation (TIPS) of a polymer solution, as previously described.^[^ ^75^, ^76^, ^77^ ^]^ To prepare the polymer solution of 2% (w/v) PLGA, 0.8 g PURASORB PDLG 7507, 75:25 DL‐lactide/glycolide copolymer (Corbion Biomaterials) was dissolved in 40 mL dimethyl carbonate (DMC) (anhydrous >99%, D152927, Sigma Aldrich) in 50 ml Falcon tubes (352 070, BD Biosciences). Samples were mixed using magnetic stirring overnight at room temperature (20 °C).

The polymer solutions were passed into Var D Nisco encapsulation unit (Nisco Engineering) and ejected through a sapphire tipped nozzle with a 150 µm opening using a syringe pump at a flow rate of 2.2 ml/min. The nozzle vibrated at a frequency of 1.80 kHz with 70% frequency amplitude. Resulting polymer droplets were collected in liquid nitrogen to induce phase separation. The samples underwent lyophilisation for 18 hours. Batches of the lyophilised TIPS microcarriers were sieved to <250 µm diameter.

### Pre‐conditioning of TIPS microcarriers

500 ml Ham's F‐10 Nutrient Mix (ThermoFisher 11 550 043) was supplemented with 20% Fetal Bovine Serum (FBS) (10 500 064, Life Technologies), 1% L‐Glutamine, 1% Antibiotics/Antimycotics (A5955, Thermo Fisher), 48 µl Dexamethasone (D4902, Sigma‐Aldrich) and 120 µl of 50 µg/ml stock Human FGF‐basic (154 a.a.) (100‐18B‐100UG, PeproTech).

A screen of pre‐conditioning solutions for the hydrophilisation of TIPS microcarriers was prepared, composed of:

(1) 5 ml Essential 8 media (A1517001, Thermo Fisher) and 1 ml 70% ethanol (E10600/05, Fisher Scientific)
(2) 5 ml complete Essential 8 media and 1 ml 70% ethanol
(3) 5 ml complete Ham's F10 media and 1 ml 70% ethanol
(4) 5 ml complete Ham's F10 media 1 ml 70% ethanol and 5 µg/ml xeno‐free Vitronectin (VTN‐N) recombinant human protein (A14700, Thermo Fisher)
(5) 5 ml HBSS, 1 ml 70% ethanol and 2.5‐20 µg/ml VTN‐N
(6) 5 ml HBSS, 1 ml 70% ethanol and 5 µg/ml albumin bovine serum (BSA) (A7030‐100G, Sigma‐Aldrich)

20 mg of TIPS microcarriers were added to the pre‐conditioning solution, vortexed for 10–15 seconds and then incubated in a hybridization oven for 72 hours – 14 days at 37 °C under constant rotation at 30 RPM.

### Scanning electron microscopy

TIPS microcarriers were mounted on aluminium discs using adhesive carbon tabs, coated with 1–2 nm of gold/palladium compound for 3 minutes in the Argon chamber of a high‐resolution ion beam coater (Gatan model 681). The microcarriers were then analysed with a Hitachi S3400N scanning electron microscope at a range of magnifications (x400 – x5000).

### Confocal microscopy

200 µl of Cellularised TIPS microcarrier suspension were stained and imaged in 8 well chambered coverglass with a working volume of 200 µl (155409PK, Thermo Fisher). The cellularised samples were washed twice with Dulbecco's phosphate buffer saline (DPBS) (D8357, Sigma), fixed using 4% paraformaldehyde solution for 10 minutes at room temperature (20 °C), washed twice with DPBS and permeabilised with 0.1% Triton (T8787, Sigma‐Aldrich) in DPBS, for 5 minutes at room temperature (20 °C). The permeabilization solution was discarded, the samples were washed thrice with DPBS and blocked for 30 minutes at room temperature (20 °C) using a 1% BSA solution in DPBS. The distribution of cell attachment on the TIPS microcarriers was evaluated using Phalloidin 633 (A12380, Thermo Fisher) and NucBlue Fixed Cell Stain (R37606, Thermo Fisher) at 1:40 and 1 drop:1 mL concentration respectively. The presence of pluripotent markers SOX2 and TRA‐1‐60 was assessed using pluripotent stem cell 4‐marker immunocytochemistry kit (A24881, Thermo Fisher) following the manufacturer's instructions. The presence of cardiac markers was assessed using the protocol described by Burridge et al., staining with cardiac troponin (Ab45932, Abcam) and Nkx2.5 (Ab91196, Abcam) antibodies, at 1:400 and 1:200 respectively in 3% BSA blocking solution incubated overnight at 4 °C. Following primary antibody staining the samples were washed thrice with DPBS and further stained with Alexa Fluor 488 (A11008, Thermo Fisher) and Alexa Fluor 594 (A21125, Thermo Fisher) secondary antibodies at 1:1000 for 1 hour at room temperature. The viability of injected iPSC‐CM on TIPS microcarriers was assessed by staining with the Apoptosis/Necrosis Assay Kit (ab176750, Abcam) and Cell mask orange stain (C10045, Invitrogen) according to the manufacturer's instructions. The samples were filled in DPBS and stored at 4 °C in the dark, for confocal imaging on the next day. Confocal microscopy was performed using Zeiss LSM 980 confocal with Airyscan 2 equipped with Zen Blue software. Images were processed using Fiji software.

### Induced‐pluripotent stem cell culture

Human episomal iPSC line was purchased from Thermo Fisher (A18945, Thermo Fisher). Cells were maintained in in Essential 8 medium and for all experiments iPSC between passages 4–10 were used. iPSC were cultured in in 6 well tissue culture plates coated with 5 µg/ml VTN‐N according to the manufacturer's instructions. Cell growth was monitored using light microscopy and passaged with Tryple (12 605 010, Thermo Fisher) once they reached 70–85% colony confluency. A split ratio of 1:3 −1:5 was used dependent on colony appearance and growth rate. After splitting, iPSC were cultured with RevitaCell Supplement (A26445, Thermo Fisher) at 1X final concentration overnight and media was replaced daily. Cell count and viability was determined using the NucleoCounter NC‐200 automated cell counter (Chemometec) according to manufacturer's instructions.

### Differentiation of induced‐pluripotent stem cells into cardiomyocytes

iPSC‐CM were obtained by differentiation of iPSC at 30% colony confluency using PSC Cardiomyocyte Differentiation Kit (A29212‐01, Thermo Fisher). Contracting cardiomyocytes were visible from day 6–8 of differentiation and maintained in culture using cardiomyocyte maintenance medium up to 40 days. For iPSC‐CM differentiation on TIPS microcarriers, iPSC (day 0 of differentiation), partially differentiated iPSC‐CM (day 2 or 4 of differentiation), early iPSC‐CM (day 6 differentiation) and late iPSC‐CM (day 18 of differentiation) were attached to the microcarriers. After cell attachment, the differentiation process was continued, by change of media, up to day 20.

### Cardiomyocyte purification

After cardiac differentiation, iPSC‐CM were isolated using the PSC‐derived CM Isolation Kit (130‐110‐188, Miltenyi Biotec) and LS separation columns (130‐042‐401, Miltenyi Biotec). iPSC‐CMs were washed twice with DPBS and dissociated using the Multi Tissue Dissociation Kit 3 (130‐110‐204, Miltenyi Biotec) as per the manufacturer's instructions. Cell suspensions were resuspended in 20% EB media (Knockout DMEM (10 829 018, ThermoFisher), 20% Hyclone‐defined FBS (10 703 464, ThermoFisher), 1:100 diluted stock non‐essential amino acids (11 140 035, ThermoFisher), 1:100 diluted stock L‐glutamine, 45.4 µM β‐Mercaptoethanol (31350‐010, ThermoFisher), 1:100 diluted stock Penicillin/Streptomycin) and filtered using a 70 µm cell strainer (CLS431751, Corning) into a 50 ml tube. Cell counting was performed to determine isolation cocktail volumes. Isolation was performed following the manufacturer's instructions using a QuadroMACS separator (130‐091‐051, Miltenyi Biotec). The enriched iPSC‐CM were cultured in pre‐warmed (37 °C) EB 2% media (DMEM (D5796, Sigma), 2% standard FBS, 1:100 diluted stock non‐essential amino acids, 1:100 diluted stock L‐glutamine, 45.4 µM β‐Mercaptoethanol, 1:100 diluted stock Penicillin/Streptomycin) supplemented with 1x RevitaCell. Cell counts were performed, and the suspension was diluted to a concentration of 100000 cells/200 µl for monolayer attachment or 600000 cells/500 µl for microcarrier attachment.

### Cell attachment and detachment to TIPS microcarriers

The pre‐conditioned TIPS microcarriers were washed with DPBS and transferred into a 24 well low bind cell culture plate (CLS3527, Sigma). 5 × 10^5^ iPSC or iPSC‐CM were suspended in 1 mL supplemented Essential 8 or cardiomyocyte maintenance media respectively and added to each well containing the microcarriers. The plate was incubated at 37 °C 5% CO_2_ on a plate shaker, shaking intermittently for 10 seconds every hour at low speed (∼50 rpm), for 24 hours.

Cellular detachment from the microcarriers was performed using TrypLE (12 605 010, Thermo Fisher). For iPSC‐CM later than 15 days of differentiation, 0.5 U/mL Liberase TH (0 540 115 1001, Roche) and 50 U/mL DNase I (18047‐019, Life Technologies) were added to the TrypLE dissociation solution to break down deposited collagen. Samples were incubated with the dissociation solution at 37 °C 5% CO_2_ for 5 minutes, twice, and larger aggregates were disrupted in between incubations by pipetting. Samples were filtered through a 70 µm cell strainer (352 340, Scientific Laboratory Supplies) to separate the cells from the microspheres.

### iPSC proliferation on TIPS microcarriers

1.00 × 10^5^ and 0.30 × 10^5^ iPSC were seeded in the well of a 5 µg/ml VTN‐N coated 6‐well plate (2D control) or 20 mg of TIPS microcarriers respectively. Seeding densities were normalised to the surface area of the substrate (9.6 cm^2^ for 2D control and 2.9 cm^2^ for microcarriers). Cellular proliferation was further monitored for a total of 7 days post‐seeding. Cell counts were taken daily at the same time using the NucleoCounter NC‐200 automated cell counter.

### Quantification of cell attachment to TIPS microcarriers

The microcarriers were washed twice with DPS to remove non‐attached cells. Using a 1 ml Pasteur pipette, the contents of each well were transferred into a 1.5 ml microfuge tube (E1415‐1500, StarLab). The microspheres were allowed to settle for 1 minute. The supernatant was removed. 300 µl of lysis buffer (Reagent A) (910‐0003, ChemoMetec) were added to the sample and vortexed for 5 seconds to mix. The solution was left to incubate for 1 min at room temperature. 300 µl of stabilization buffer (Reagent B) (910‐0002, ChemoMetec) were added to the sample and vortexed for 5 seconds to mix. 300 µl of fresh, Essential 8 media or CM maintenance media, was added to the 1.5 ml microfuge tube containing the microcarriers. 200 µl of cell suspension was loaded into ChemoMetec Via1‐Cassette. The cassette was loaded into the NucleoCounter NC‐200 automated cell counter, and cell count and viability were determined.

### Evaluation of cell attachment and migration on iPSC pluripotency

To evaluate the effect of microcarrier interaction on iPSC pluripotency under conditions that simulated pre‐ and post‐ cellularisation of the microcarriers, iPSC attached to TIPS microspheres and migrated off TIPS microspheres were analysed. iPSC attachment to TIPS microspheres was analysed after 24 hour of seeding. For iPSC migration, iPSC were migrated off the TIPS microspheres and onto a 5 µg/ml VTN‐N coated 8 well chambered slide for confocal or 5 µg/ml VTN‐N coated tissue culture plate for flow cytometric analysis. The spent media was removed and replaced with fresh Essential 8 media and the samples were maintained in culture for 72 hours. Migration off the TIPS microsphere and onto 5 µg/ml VTN‐N coated 2D surface was monitored using a light microscope.

Controls included a 2D control of iPSC cultured on 5 µg/ml VTN‐N coated tissue culture plastic, an attachment control and a migration control, matching the length of culture and passage number to the samples on TIPS microcarriers. Specifically, the attachment control consisted of iPSC passaged once, with re‐attachment onto 5 µg/ml VTN‐N coated tissue culture plastic for 24 hours. The migration control consisted of iPSC passaged once onto VTN‐N coated tissue culture plate for 24 hours, following a second passage onto fresh VTN‐N coated tissue culture plate for 72 hours.

Samples were washed twice with DPBS to remove unbound cells and analysed by flow cytometry and confocal microscopy.

### Western Blotting

Suspension samples were lifted using Tryple or cardiomyocyte dissociation solution and resuspended in a 6‐well low bind plate for 1, 2, 4, 8 or 24 hours. Positive control samples were treated with 2 ml of complete E8 or cardiomyocyte maintenance media supplemented with 100 µM etoposide (E1383‐25MG, Sigma‐Aldrich) for 4 hours. Suspension and microcarrier samples were transferred from the low‐binding plates into 1.5 ml microfuge tubes and centrifuged at 200 G for 5 minutes. Diluents were discarded and the samples were snap frozen on dry ice. Proteins were extracted using a lysis buffer composed of Radioimmunoprecipitation assay (RIPA) buffer (R0278, Sigma‐Aldrich) complimented with protease and phosphatase inhibitor (A32961, Thermo Scientific). The eluted protein was transferred into a new microfuge tube kept on ice. The total protein concentration was quantified using the Pierce Bicinchoninic (BCA) assay (23 225, Thermo Scientific) according to the manufacturer's instructions. Boiled samples and 15 µl of pre‐stained protein ladder (PL00001, Protein‐Tech) were separated by SDS‐PAGE and subsequently electro‐transferred onto polyvinylidene fluoride (PVDF) membranes (LC2002, Thermo Scientific) through wet transfer using Bolt transfer buffer (BT00061, Thermo Scientific). The protein‐loaded membranes were blocked with a blocking solution, composed of 5% (w/v) non‐fat dried milk in 1:10 diluted 10x Tris Buffered Saline with Tween 20 (TBST) (12498S, Cell Signalling Technology), for 1 hour at room temperature on a rocking platform. The membranes were incubated with primary antibodies overnight at 4 °C on a rocking platform. Antibodies were diluted in blocking solutions as described in Table S1. After TBST washing, the membranes were incubated with horseradish peroxidase (HRP)‐conjugated secondary antibodies diluted in blocking solution, for 1 hour at room temperature. The membranes were washed and developed using Pierce ECL Western Blotting Substrate (32 209, Thermo Scientific) exposed to autoradiography films (34 090, Thermo Scientific). The developed films were scanned at 600 dpi and densitometry analysis was performed using FIJI software. The quantified protein bands were normalised against the loading control GAPDH and the protein of interest expression was displayed as an intensity ratio relative to the loading control.

### Flow cytometry

Prior to flow cytometry, cell populations were washed twice with DPBS to remove unbound cells and isolated from 2D tissue culture vessels or TIPS microcarriers. Samples were kept on ice for the length of the staining protocol.

Flow cytometry was used to assess the cellular phenotype of iPSC and iPSC‐CM using the antibodies listed in Table S2. After Live/Dead staining, samples were fixed with 4% PFA, stained for extracellular markers at 4 °C for 30 minutes in the dark. The samples were permeabilised by washing twice and staining with 1X intracellular staining permeabilization wash buffer (421 002, Biolegend). Staining for intracellular markers was performed in the permeabilization wash buffer. The samples were stained 4 °C for 30 minutes in the dark. The samples were washed twice with DPBS and resuspended in 300 µl of DPBS in FACS tubes (352 054, Falcon) ready for analysis.

Samples were analysed on a BD LSR Fortessa I machine. All flow cytometry data was analysed using FlowJo software (Tree Star Inc.). Gates were set based on fluorescence minus one (FMO) stains. Compensation controls were based on single‐stained samples for each antibody in the panel and used to create a fluorochrome compensation matrix.

### Sample preparation and trilineage differentiation for qPCR

Control P7 iPSC samples were cultured in 5 µg/ml VTN‐N coated 6 well tissue culture plates and harvested after reaching 70–80% colony confluence. Microcarrier interaction was investigated by attaching 5 × 10^5^ P7 iPSC to 20 mg of 2% 7507 TIPS microcarriers pre‐conditioned with 5 µg/ml of VTN‐N, overnight. Trilineage differentiated cells were obtained from iPSC cultured in 6 well plates at 30–40% culture confluence. iPSC were differentiated into endoderm progenitor cells using the method described by Ishikawa et al.^[^ ^78^ ^]^ Endoderm differentiation media was made of Advanced RPMI 1640 media (12 633 012, Thermo Fisher) supplemented with 1% antibiotic antimycotic solution (A5955, Sigma‐Aldrich), 1% Gluatamax (35 050 061, Thermo Fisher) and 0.2% FBS (10 500 064, Life Technologies). Differentiation towards endoderm lineage was performed by replacing supplemented E8 media with 2 ml of endoderm differentiation media supplemented with 50 ng/ml activin A (120‐4P, Peprotech) and 5 µM CHIR‐99021 (1046‐5MG, Sigma Aldrich) for 24 hours, followed by a 24‐hour treatment with the same medium without CHIR‐99021. Ectoderm differentiation was performed by the method described by Tchieu et al.^[^ ^79^ ^]^ Ectoderm basal differentiation media was made of 8.2 ml DMEM (41966‐029, Gibco), 1.5 ml knockout serum replacement (10 828 028, Thermo Fisher), 100 µl antibiotic antimycotic solution (A5955, Sigma‐Aldrich), 100 µl Glutamax, 100 µl MEM non‐essential amino acids (11 140 050, Thermo Scientific) and 10 µl 2‐mercaptoethanol (21 985 023, Thermo Fisher). Differentiation towards ectoderm lineage was performed by replacing supplemented E8 media with 2 ml of ectoderm differentiation media supplemented with 500 nM LDN193189 (1066208‐5MG, Biogems) and 10 µM SB431542 (3014193‐1MG, Biogems) for 24 hours. The media was replaced with 2 ml fresh differentiation media supplemented with LDN193189 and SB431542 for an additional 24 hours. For mesoderm differentiation, supplemented E8 media was replaced with 2 ml of Media A for 48 hours.

### qPCR

Oligonucleotide primers were designed using Primer Blast, to produce a PCR product size of 70–150 base pairs, predesigned to span or flank introns, anneal at 60 °C, <55% GC content and synthesised on a 25 molar scale by Thermo Fisher (Table S3). Primer efficiency was checked by producing a standard curve of Ct value versus cDNA concentration per primer pair. Additionally, specificity was tested by amplicon melt curve analysis, by observing for a single peak to confirm specific primer annealing. RNA extraction was performed using the RNeasy Mini Kit (Qiagen) according to the manufacturer's instructions. The extracted RNA was reverse transcribed into cDNA using Quantabio qScript cDNA Supermix (733‐1177, VWR). qPCR reactions were set to run in a MircoAmp Optical 96‐well reaction plate (10 411 785, Fisher Scientific) using Brilliant II SYBR Green QPCR (600 882, Agilent), according to the manufacturer's instructions. Gene expression measurement was assessed using an Eppendorf Mastercycler ep Gradient S sequence detection system. Gene of interest expression was normalised to the endogenous housekeeping gene GAPDH. Relative gene expression was calculated in absolute values of 2^−∆Ct^.

### Delivery formulation and in vitro injectability studies

TIPS microcarriers were suspended into gel formulation in order to produce a homogenous microcarrier suspension for in vivo delivery. To select the formulation, 0.1 cm^3^ of 2% 7507 pre‐conditioned TIPS microcarrieres were overlaid on the surface of 1 ml dilutions of 20–60% (v/v) GranuGel (Convatec) in dH_2_O, and time for microcarrier sedimentation was measured.

For in vitro injectability studies, 1 × 10^6^ purified day 18 iPSC‐CM were seeded on 20 mg of microcarriers and attached under static dynamic conditions for 24 hours. The media was replaced, and the cells were left to recover for another 72 hours. 4 days after cell seeding, the sample was resuspended in 600 µL of 60% GranuGel. 200 µl of 60% GranuGel and microcarriers suspension were loaded in a 1 ml B Braun Injekt‐F fine dosage syringe (10 303 002, Fisher Scientific), and capped with a 23G/20 mm/pt style 4 needle (RE55167,Thames Restek) or left uncapped. The solution was injected into a 24 well low bind plate and the injected microspheres were immediately analysed or cultured up to 6 days. 2 mL of EB 2% media was added to the cultured samples and media was replaced every two days post‐injection, up to day 6.

### iPSC‐CM spontaneous activity recording

iPSC‐CM spontaneous contractions were recorded using a brightfield microscope. For each independent biological sample, 4 different regions were recorded at 60 frames per second (fps) for 30 seconds. Spontaneous activity was defined as spontaneous beating frequency (number of contractions/recording time).

### Optical mapping

The samples used for optical mapping were obtained from 6 independent batches of differentiation and pooled into 3 independent purification batches. The Ca^2+^ transients of purified iPSC‐CM attached to TIPS microcarriers versus iPSC‐CM in monolayer on 35 mm plastic dishes (150 460, Thermo Fisher) were compared using high resolution optical mapping.^[^ ^80^ ^]^ Suspension iPSC‐CM attached to TIPS microcarriers were transferred to 35 mm plastic dishes for optical mapping. Samples were loaded with Ca^2+^ dye Fluo‐4AM (F14201, Thermo Fisher) (4 µM plus 1 mM Probenecid (P36400, Thermo Fisher) in DMEM for 20 min followed by 20 min de‐esterification in fresh DMEM). iPSC‐CM were superfused with Normal Tyrode's solution (140 mM NaCl, 4.5 mM KCl, 10 mM glucose, 10 mM 4‐(2‐hydroxyethyl)−1‐piperazineethanesulfonic acid, 1 mM MgCl2, 1.8 mM CaCl2, pH 7.4) pre‐warmed at 37 °C. Optical mapping was performed with a Photometrics Evolve 512 EMCCD camera (Photometrics) mounted on a custom macroscope (Cairn Research) equipped with a water‐immersion 20x objective, appropriate excitation/emission filters, and 470 nm light‐emitting diode illumination. Data was acquired using the recording software WinFluor.^[^ ^81^ ^]^ Calcium transient recordings were performed at 200 fps, from an 820 × 820 µm area. The acquired data was analysed using OPTIQ software (Cairn Research) and filtered using a Gaussian spatial filter (radius 2 pixels) before relevant parameters were extracted.^[^ ^82^ ^]^ 5 regions of interest were analysed for each biological replicate (n = 3‐11 independent biological replicates).

### Statistical analysis

Data reported was collected from technical and biological replicates with samples sizes indicated in figure legends. Data are presented as mean and standard deviation (±SD). Prior to statistical analysis, the data was first tested for normality through a Shapiro‐Wilk test. Statistical significance for difference between experimental groups was determined using a Student's t‐test. Analysis of statistical significance for two groups or more was performed using ANOVA followed by post‐hoc test. p values of <0.05 indicated statistical significance and were shown as p ≤ 0.05 = *, p ≤ 0.01 = **, p ≤ 0.001 = *** and p ≤ 0.0001 = ****. The data were plotted using GraphPad Prism 8 Software (GraphPad Holdings LLC).

## Conflict of Interest

The authors declare no conflict of interest.

## Supporting information

Supporting Information

Supplemental Video 1

Supplemental Video 2

Supplemental Video 3

## Data Availability

Data supporting this study are included within this article and supporting materials. Full data are available from the corresponding author upon reasonable request.
