# Supplementary material for: Injectable biodegradable microcarriers for iPSC expansion and cardiomyocyte differentiation
Source: Adv Sci (Weinh). 2024 Jun 20;11(32):2404355. doi: 10.1002/advs.202404355 (PMC11348074; doi:10.1002/advs.202404355)
Supplement: Supplementary file 1 — Supporting Information [file ADVS-11-2404355-s001.pdf]

ADVANCED SCIENCE
Open Access

Supporting Information

for *Adv. Sci.*, DOI 10.1002/advs.202404355

Injectable biodegradable microcarriers for iPSC expansion and cardiomyocyte differentiation

Annalisa Bettini, Patrizia Camelliti, Daniel J. Stuckey and Richard M. Day**

Supporting Information

Injectable biodegradable microcarriers for iPSC expansion and cardiomyocyte differentiation

Annalisa Bettini*, Patrizia Camelliti, Daniel J. Stuckey and Richard M. Day*

Video 1: Beating iPSC-CM on TIPS microcarriers after 7 days in culture

Video 2: Beating iPSC-CM on TIPS microcarriers after 10 days in culture

Video 3: Beating iPSC-CM on TIPS microcarriers after 14 days in culture

Type	Antibody	Species raised in	Catalogue number	Supplier	Dilution
Primary	Caspase-3 (3G2) Mouse mAb	Mouse	9668S	Cell Signalling	1:1500
Primary	Cleaved Caspase-3 (Asp175) Antibody	Rabbit	9661S	Cell Signalling	1:750
Primary	Caspase-9 (C9) Mouse mAb	Mouse	9508S	Cell Signalling	1:1000
Secondary	Goat anti-rabbit IgG (H+L) HRP conjugate	Goat	SA00001-2	Protein-Tech	1:5000
Secondary	Goat anti-mouse IgG (H+L), HRP conjugate	Goat	SA00001-1	Protein-Tech	1:2000
Primary loading control	GAPDH Mouse McAb	Mouse	60004-1-Ig	Protein-tech	1:10’000

Table S1: Western blot antibodies.

Antibody	Fluorochrome	Target location	Catalogue ID	Supplier	Concentration
LIVE/DEAD™	Near-IR	/	L10119	Invitrogen	1:1000
iPSC Panel					
SSEA-4	PE/Cyanine7	Extracellular	330420	Biolegend	1:20
TRA-1-60	FITC	Extracellular	560380	BD	Pre-diluted at recommended volume
OCT3/4	BV510	Intracellular	563524	BD	1:20
SOX2	BV421	Intracellular	656114	Biolegend	1:50
iPSC-CM Panel					
Cardiac Troponin T	AF647	Intracellular	565744	BD	1:100
α-Actinin (Sarcomeric)	PE	Intracellular	30-123-773	Miltenyi Biotec	1:50

Table S2: Flow cytometry antibodies.

	Gene	Accession ID	Primer type	Sequence (5'->3')	Product length	Start	Stop	Melting temperature	GC%
Pluripotency	SOX2	NM_003106.4	Forward	GCTACAGCATGATGCAGGACCA	135	945	966	62.96	54.55
			Reverse	TCTGCGAGCTGGTCATGGAGTT		1079	1058	63.96	54.55
	POU5F1B	NM_001159542.2	Forward	TTCGGATTTCGCCTTCTCGC	98	60	79	61.08	55
			Reverse	GGAAGCTTAGCCAGGTCAGA		157	138	59.09	55
	NANOG	NM_024865	Forward	CTCCAACATCCTGAACCTCAGC	115	569	590	60.94	54.55
			Reverse	CGTCACACCATTGCTATTCTTCG		683	661	60.24	47.83
	PODXL	NM_005397	Forward	CCTGAACCTCACAGGAAACACC	136	1247	1268	61.07	54.55
			Reverse	TGGAACAGATGCCAGCCGTATG		1382	1361	62.95	54.55
Ectoderm	*ALDH1A0*	NM_000689.5	Forward	TGCCGGGAAAAGCAATCTGA	95	824	843	60.25	50
			Reverse	CAACAGCATTGTCCAAGTCGG		918	898	60.07	52.38
	COL1A1	NM_000088.4	Forward	GAGGGCCAAGACGAAGACATC	140	203	223	60.74	57.14
			Reverse	CAGATCACGTCATCGCACAAC		342	322	59.94	52.38
	PAX6	NM_001604	Forward	CTGAGGAATCAGAGAAGACAGGC	131	1275	1297	60.43	52.17
			Reverse	ATGGAGCCAGATGTGAAGGAGG		1405	1384	61.82	54.55
	DCX	NM_000555	Forward	TGCCTCAGGGAGTGCGTTA	94	535	553	60.91	57.89
			Reverse	GAACAGACATAGCTTTCCCCTTC		628	606	59.06	47.83
Endoderm	*AFP*	NM_001134	Forward	TGAGCACTGTTGCAGAGGAG	96	698	717	59.97	55
			Reverse	TTGTTTGACAGAGTGTCTTGTTGA		793	770	59	37.5
	GATA1	NM_002049	Forward	TGCGGCCTCTATCACAAGATG	82	757	777	60.2	52.38
			Reverse	CTGCCCGTTTACTGACAATCA		838	818	58.58	47.62
	SOX7	NM_031439	Forward	TCGACGCCCTGGATCAACT	143	1013	1031	60.98	57.89
			Reverse	CTGGGAGACCGGAACATGC		1155	1137	60.45	63.16
	CDH1	NM_004360	Forward	GCCTCCTGAAAAGAGAGTGGAAG	131	1652	1674	60.87	52.17
			Reverse	TGGCAGTGTCTCTCCAAATCCG		1782	1761	62.57	54.55
Mesoderm	*DCN*	NM_001920	Forward	GCTCTCCTACATCCGCATTGCT	128	754	775	62.75	54.55
			Reverse	GTCCTTTCAGGCTAGCTGCATC		881	860	61.32	54.55
	GATA2	NM_032638	Forward	CAGCAAGGCTCGTTCCTGTTCA	150	1197	1218	63.32	54.55
			Reverse	ATGAGTGGTCGGTTCTGCCCAT		1346	1325	64	54.55

	BMP4	NM_001202	Forward	GCACTGGTCTTGAGTATCCTG	135	502	522	58.1	52.38
			Reverse	TGCTGAGGTTAAAGAGGAAACG		636	615	58.6	45.45
	IGF2	NM_000612	Forward	AGACGTACTGTGCTACCCC	121	931	949	58.42	57.89
			Reverse	TGCTTCCAGGTGTCATATTGG		1051	1031	58	47.62
	TBXT	NM_003181.4	Forward	TGCTTCCCTGAGACCCAGTT	121	1061	1080	60.77	55
			Reverse	GATCACTTCTTTCCTTTGCATCAAG		1181	1157	58.91	40
House-keeping	*GAPDH*	NM_002046	Forward	AATGAAGGGGTCATTGATGG	108	190	171	55.35	45
			Reverse	AAGGTGAAGGTCGGAGTCAA		83	102	58.58	50

Table S3: qPCR Primers. Oligonucleotide primers were designed using Primer Blast, to produce a PCR product size of 70-150 base pairs, predesigned to span or flank introns, anneal at 60ºC, <55% GC content and synthesised on a 25 molar scale by Thermo Fisher.

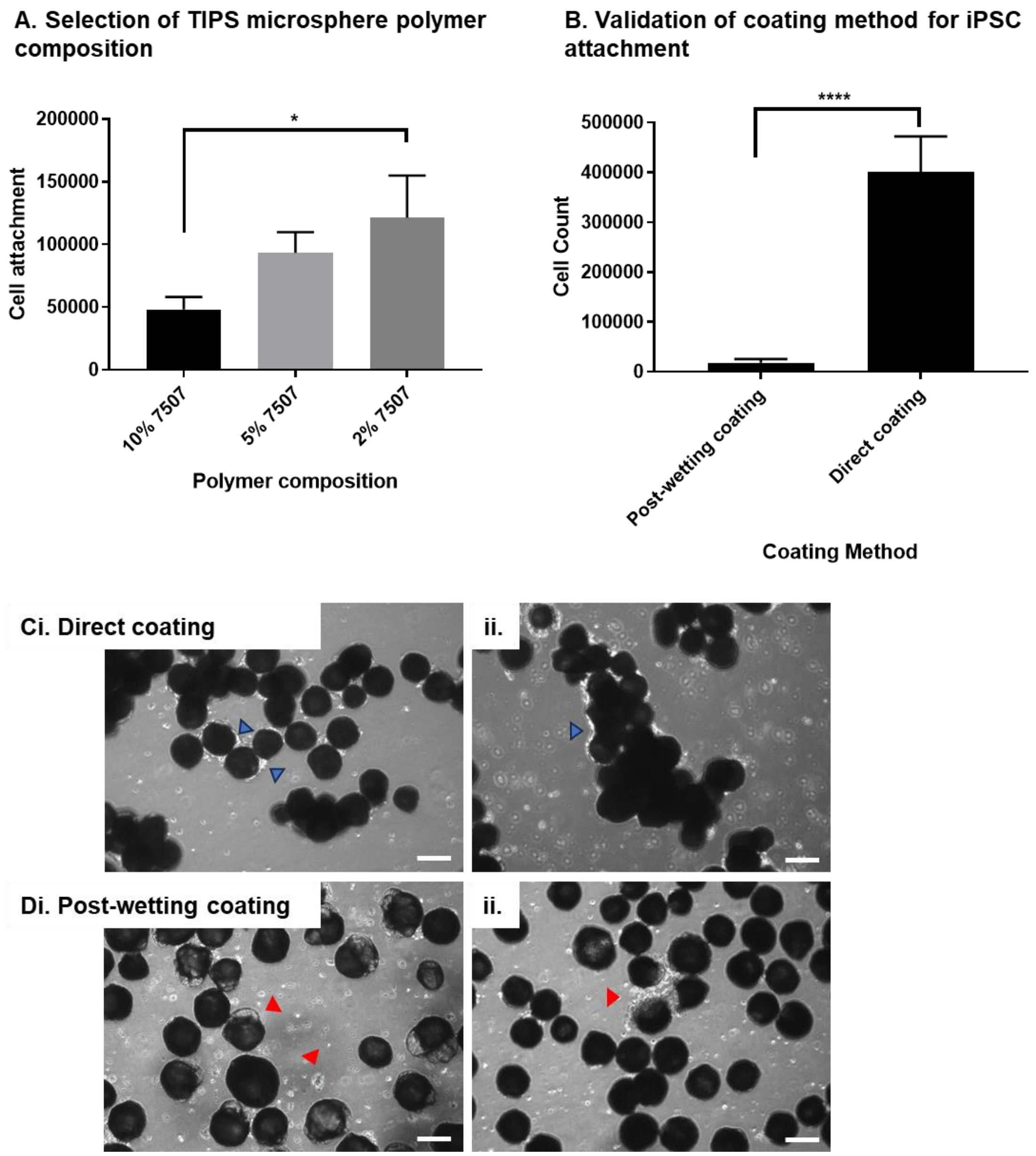

Figure S1: Selection of TIPS microcarriers PLGA composition and pre-conditioning method. (A) Quantification of iPSC attachment to 2%, 5% and 10% 7507 TIPS microcarriers. 2.5×10^5 P10 iPSC were seeded on 0.1cm^3 of microcarriers and attached under static dynamic conditions over 24 hours. (B) Comparison of direct- versus post-wetting pre-conditioning of TIPS microcarriers. 5×10^5 P7 iPSC were seeded on 0.1cm^3 of microcarriers and attached under static dynamic conditions over 24 hours. Post-wetting coating refers to exposure of the microcarriers to VTN-N, after pre-conditioning. Direct coating refers to exposure of the microcarriers to VTN-N during pre-conditioning. Representative images of iPSC attached to TIPS microcarriers coated (C) directly during the pre-conditioning process, or (D) post-conditioning. Blue arrows show cell attachment to the microcarriers, with formation of cellular connections between microcarriers. Red arrows show cellular clumping and uneven attachment of cells to the microcarriers. Scale bars represent 100 µm. Data are presented as mean ±SD. The significance of the data was calculated by (A) two-way ANOVA, Tukey’s post-hoc correction and (B) Unpaired t-test two tailed. (n=6, *P≤0.05, ***P≤0.001, ****P≤0.0001)

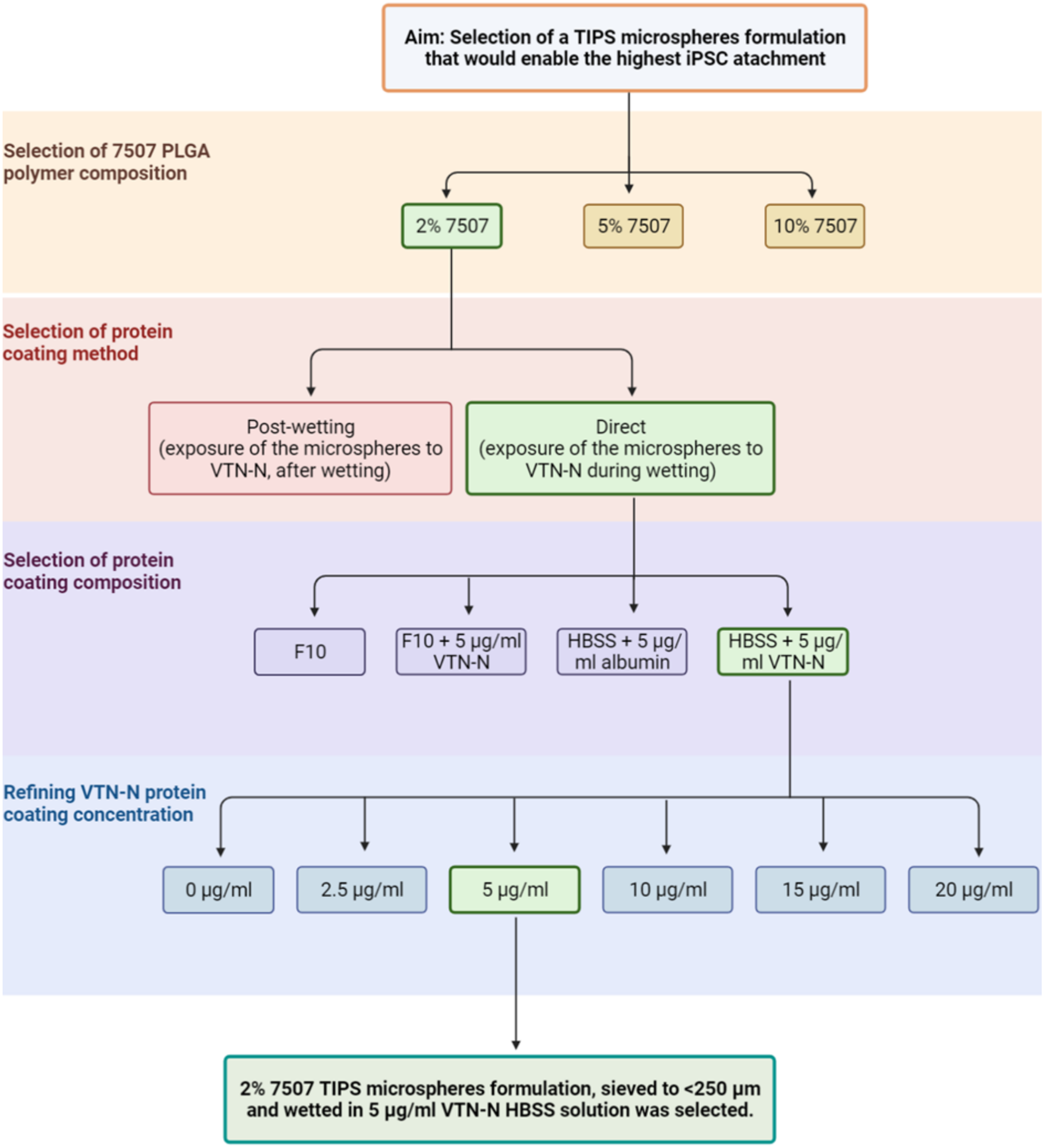

Figure S2: Experimental strategy utilised for the selection of the TIPS microcarrier formulation that would enable the highest iPSC attachment. Selected parameters shown in green boxes.

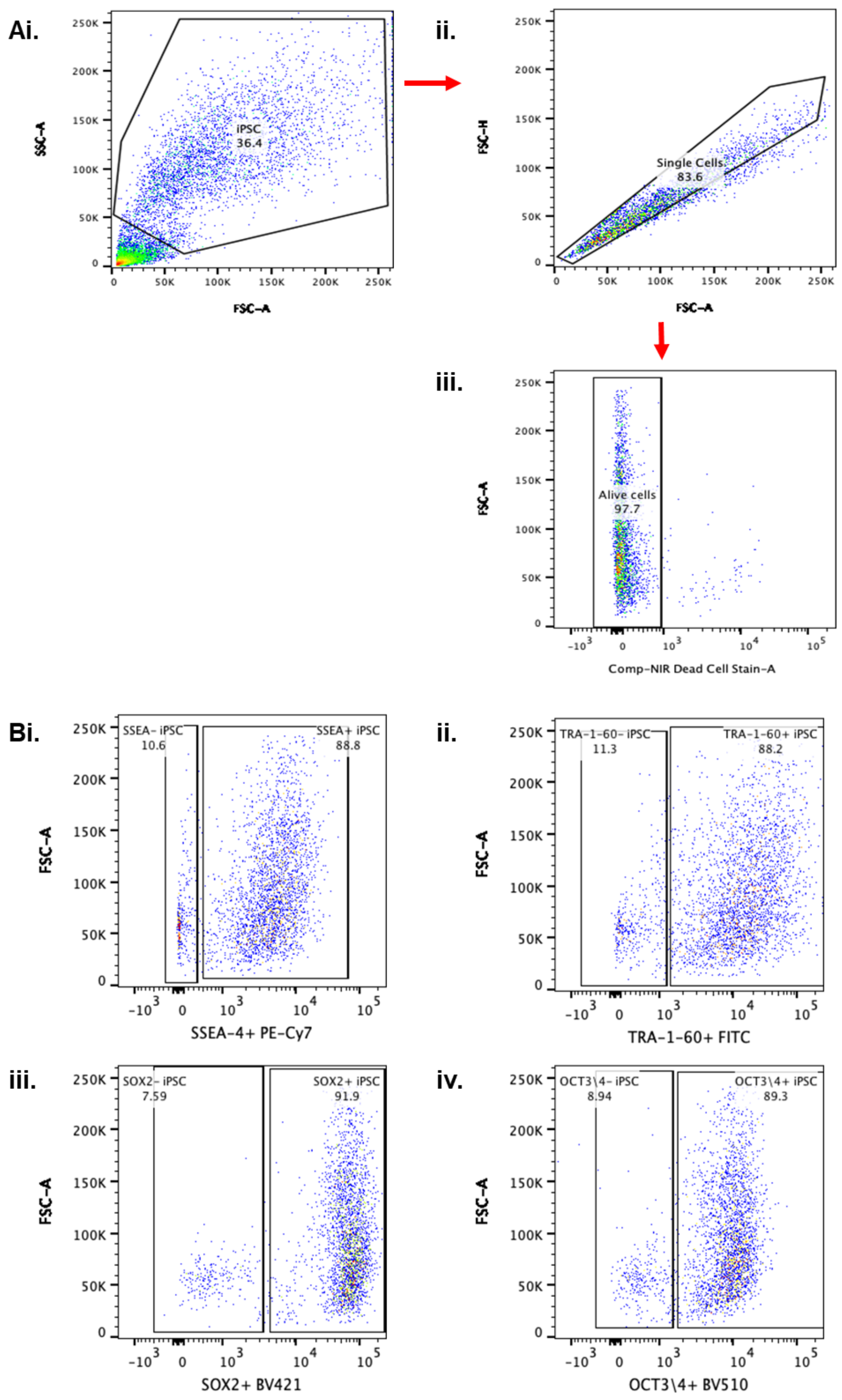

Figure S3: Gating strategy utilised to characterise iPSC populations. (A) Selection of live iPSC population by (i) side scatter vs. forward scatter area density plots to remove debris, (ii) side scatter vs. forward scatter height density plots to remove doublets, and (i) selection of live cells by negative expression of dead cell stain. (B) The live cell population was further gated to select the positive population of iPSC expressing (i) SSEA-4, (ii) TRA-1-60, (iii) SOX2 and (iv) OCT4. Representative plots showing gating set according to FMO controls and negative control cells.

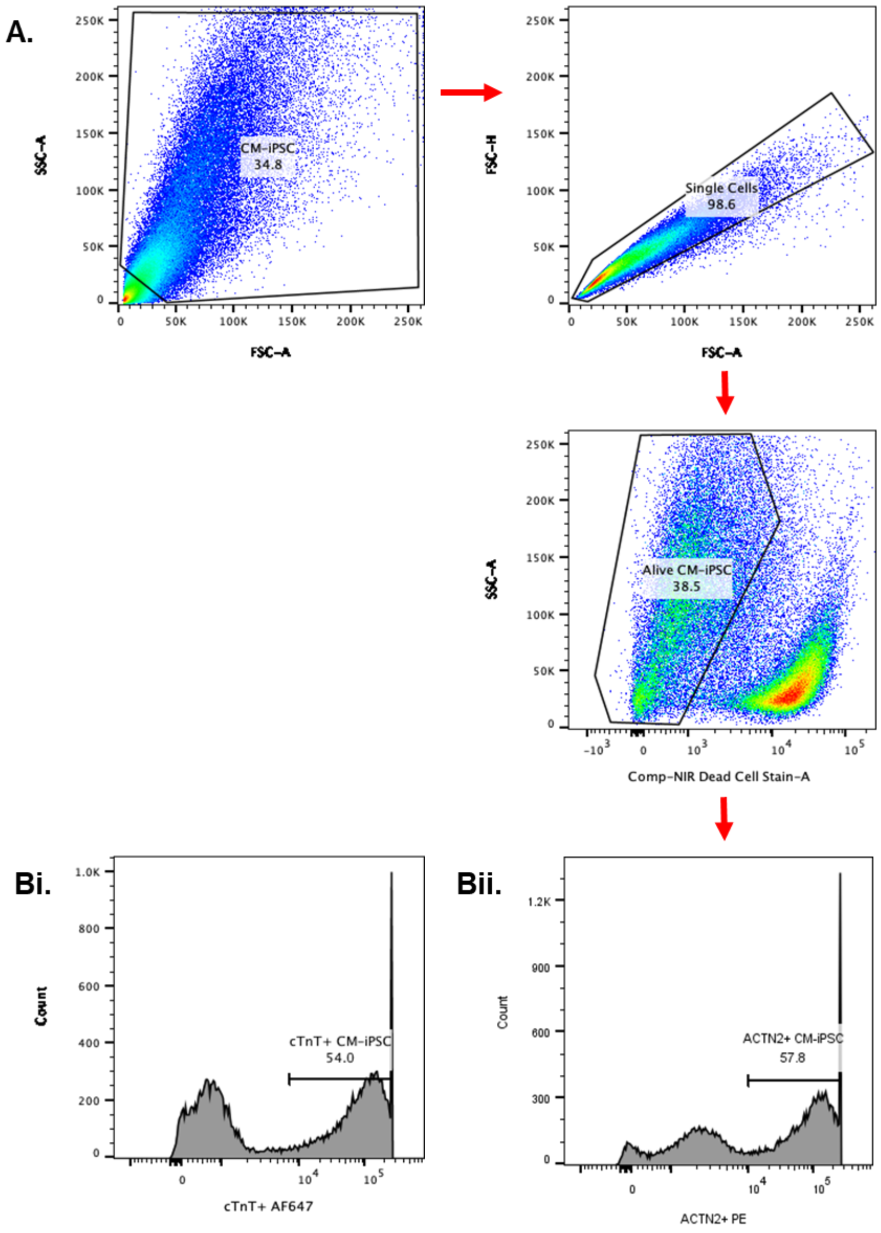

Figure S4:Gating strategy utilised to characterise iPSC-CMs population. (A) side scatter vs. forward scatter area density plots to remove debris, side scatter vs. forward scatter height density plots to remove doublets, and selection of live cells by negative expression of dead cell stain. (B) Selection of cTNT and ACTN2 positive cell population. Representative plots showing gating set according to unstained and negative control cells.

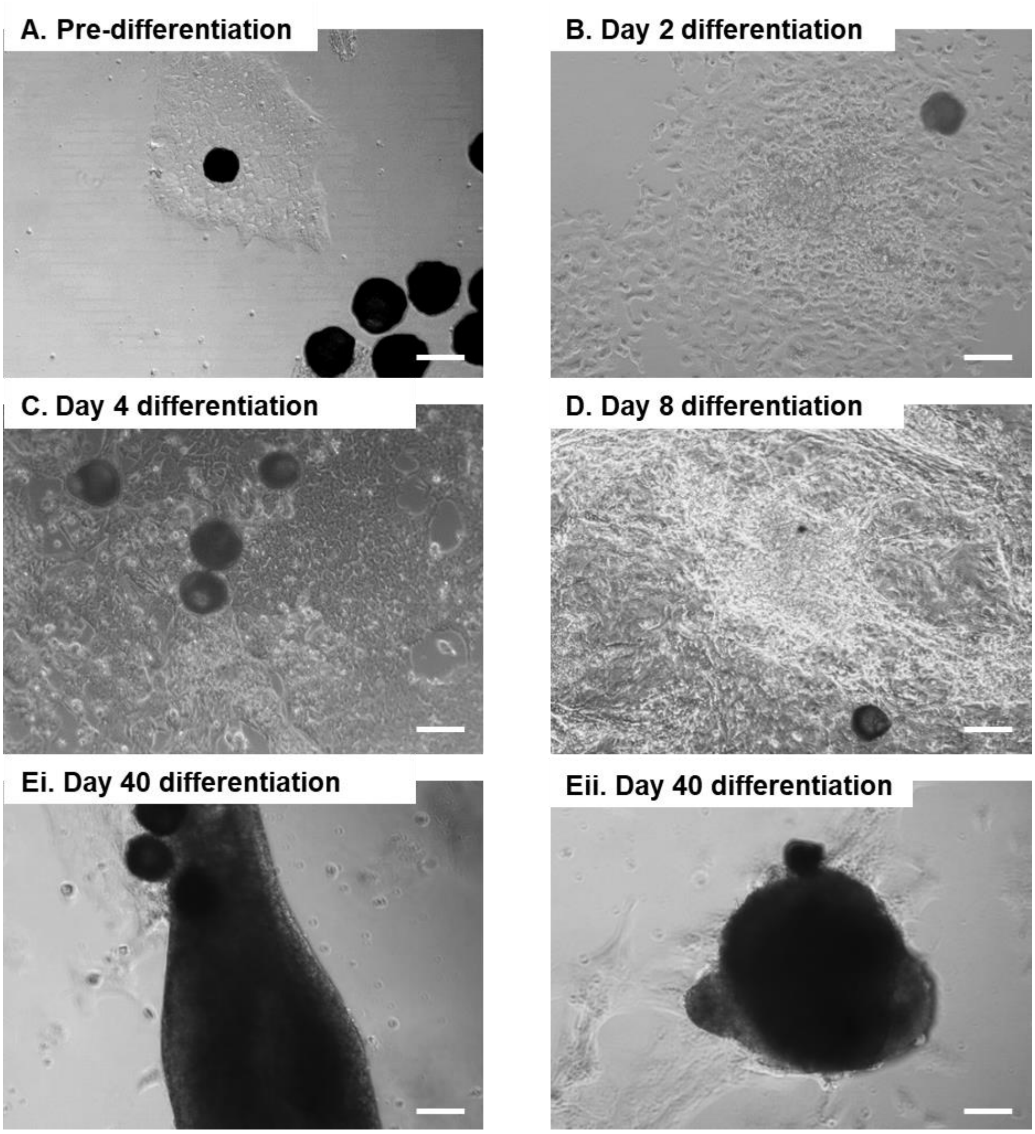

Figure S5: iPSC migration off TIPS microcarriers, and cardiac differentiation. (A) iPSC migrated off TIPS microcarriers onto VTN-N coated tissue culture plastic. Migrated iPSC were differentiated and imaged at (B) day 2 and (C) day 4 of cardiac differentiation. Beating bundles were visible at (D) day 8, and up to (E) day 40 of differentiation. Scale bar represents 100 µm.

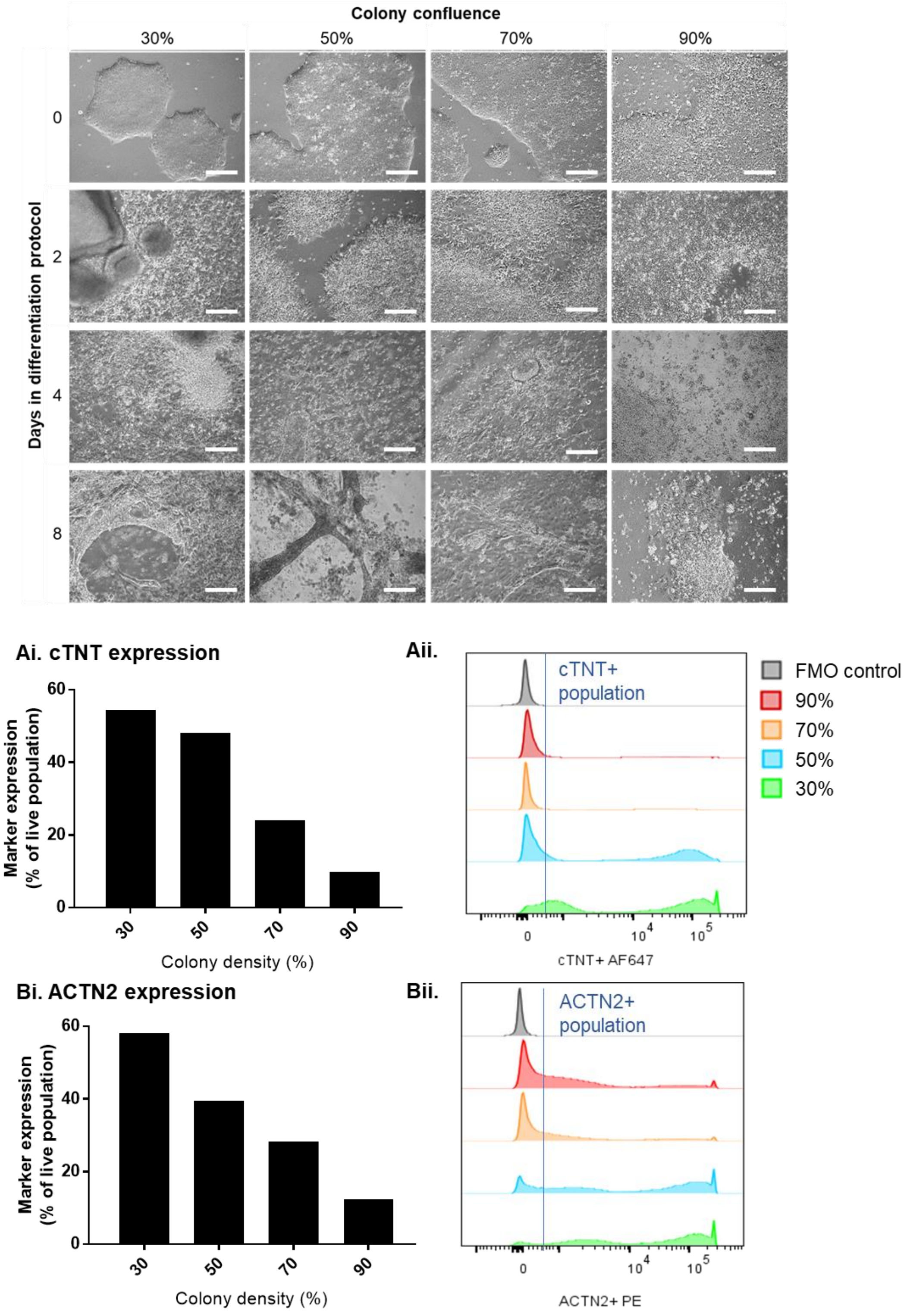

Figure S6: Impact of colony confluency on iPSC differentiation into iPSC-CM. Images of iPSC 4 days after seeding, at 30%, 50%, 70% and 90% colony confluence. Confluence measurements were performed by visual estimation. The colonies were differentiated into iPSC-CM for 10 days. Optimal confluence for cardiac differentiation was about 30-50%, where cells formed beating bundles from day 6-8 of differentiation. Cardiac differentiation efficiency was quantified by flow cytometric analysis of expression of cardiac markers (A) cTNT and (B) ACTN2. Scale bar represents 100 µm. Data are presented as mean. (n=1-2).

Western blot films for cellularised TIPS microcarrier samples

A. Caspase 3

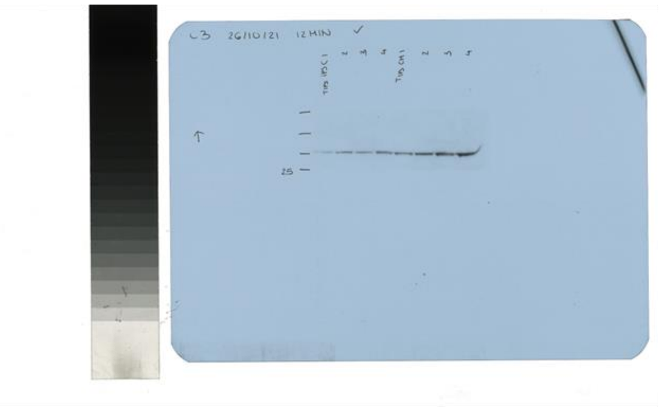

B. Caspase 9 and cleaved caspase 3

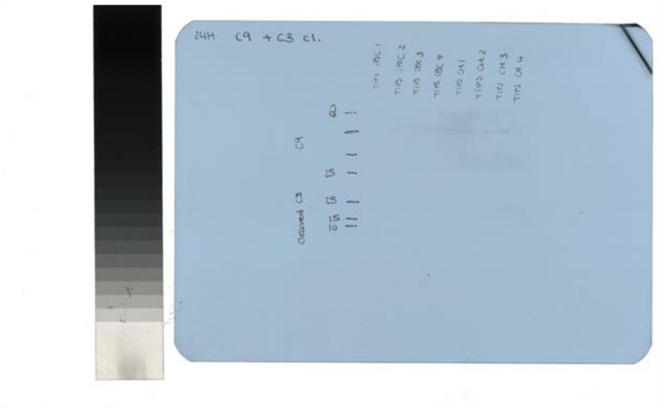

C. GAPDH

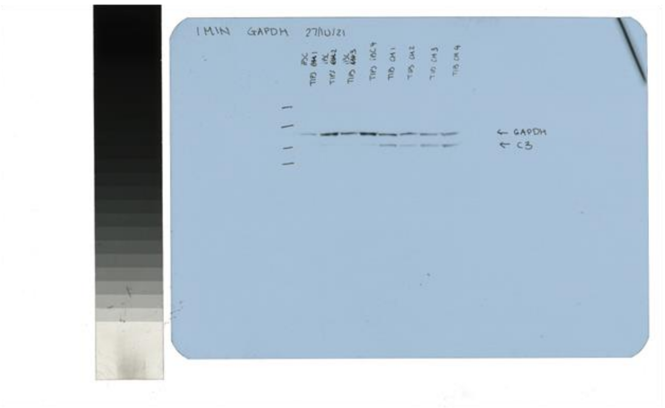

Figure S7: Raw western blot film scans used to assess anoikis activation in cellularised TIPS microcarriers samples. iPSC and iPSC-CM attached to TIPS microcarriers were characterised for the by the expression downstream markers of apoptosis, inactive caspases (A) 3 and (B) 9, active cleaved caspase 3. Protein quantification was normalised to the expression of housekeeper GAPDH. Cleaved caspase 3 (cleaved C3).

Western blot films for iPSC-CM samples

A. Cleaved caspase 3

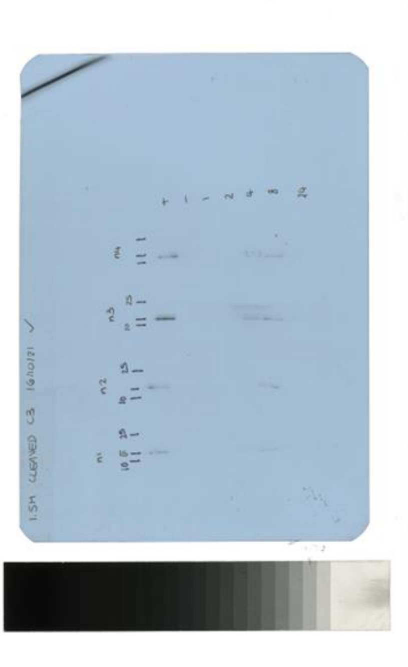

B. Caspase 3

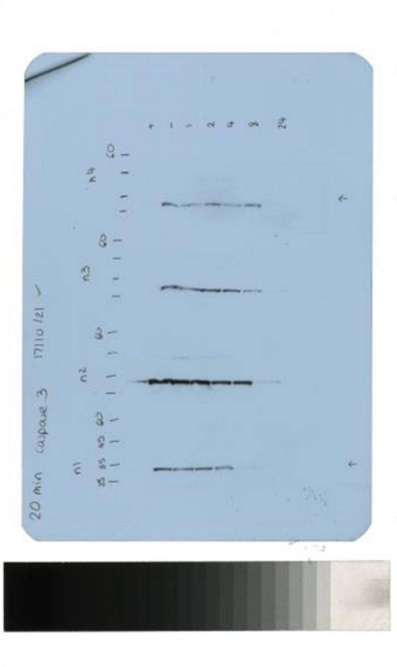

C. Caspase 9

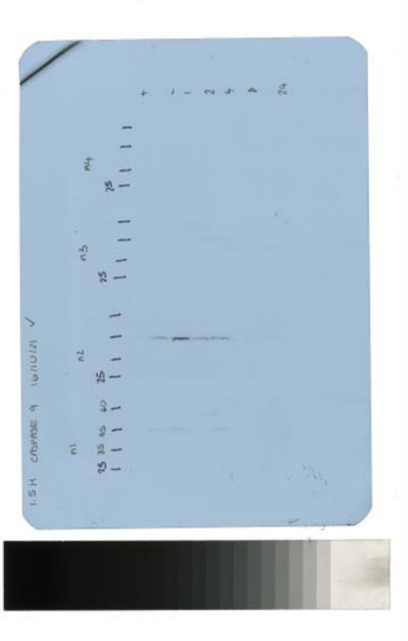

D. GAPDH

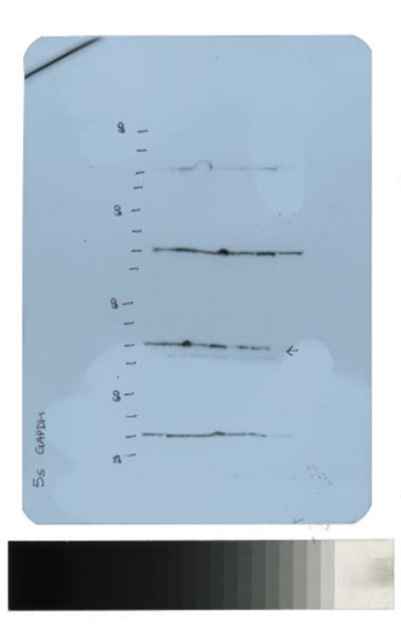

Figure S8: Raw western blot film scans used to assess anoikis activation in iPSC-CM. iPSC-CM controls and suspension samples were characterised for the by the expression downstream markers of apoptosis, inactive caspases (B) 3 and (C) 9, (A) active cleaved caspase 3. (D) Protein quantification was normalised to the expression of housekeeper GAPDH. Negative control (-) refers to cells cultured on 2D tissue culture plastic surfaces, Positive control (+) for the onset of cell death were treated with 100 µM etoposide for 4 hours, Hours (h) refers to the amount of time samples were held in suspension to induce anoikis, Cleaved caspase 3 (cleaved C3).

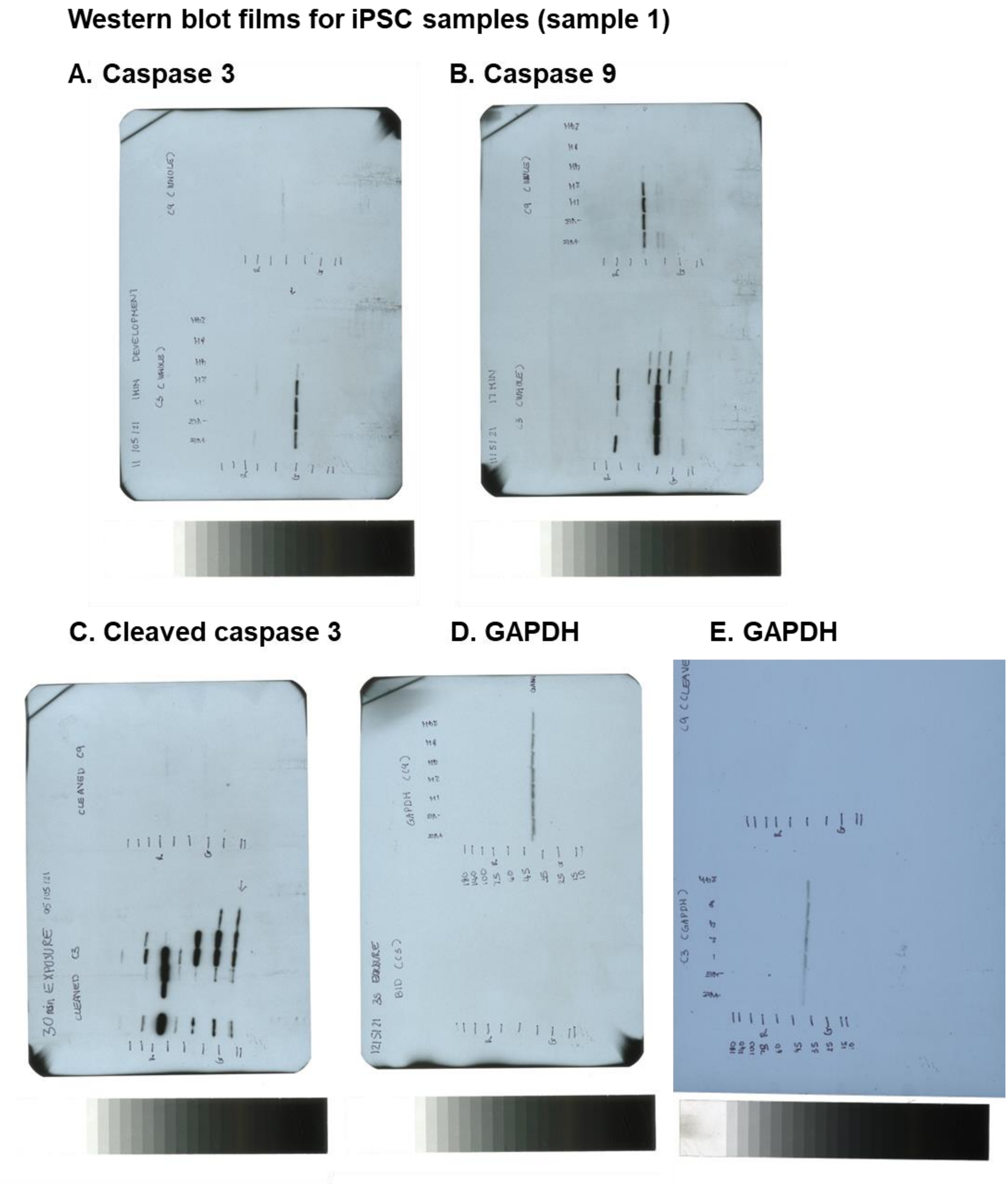

Figure S9: Raw western blot film scans used to assess anoikis activation in iPSC. iPSC controls and suspension samples were characterised for the by the expression downstream markers of apoptosis, inactive caspases (A) 3 and (B) 9, (C) active cleaved caspase 3. (D-E) Protein quantification was normalised to the expression of housekeeper GAPDH. Negative control (-) refers to cells cultured in 2D control conditions, Positive control (+) for the onset of cell death were treated with 100 µM etoposide for 4 hours, Hours (h) refers to the amount of time samples were held in suspension to induce anoikis, Cleaved caspase 3 (cleaved C3).

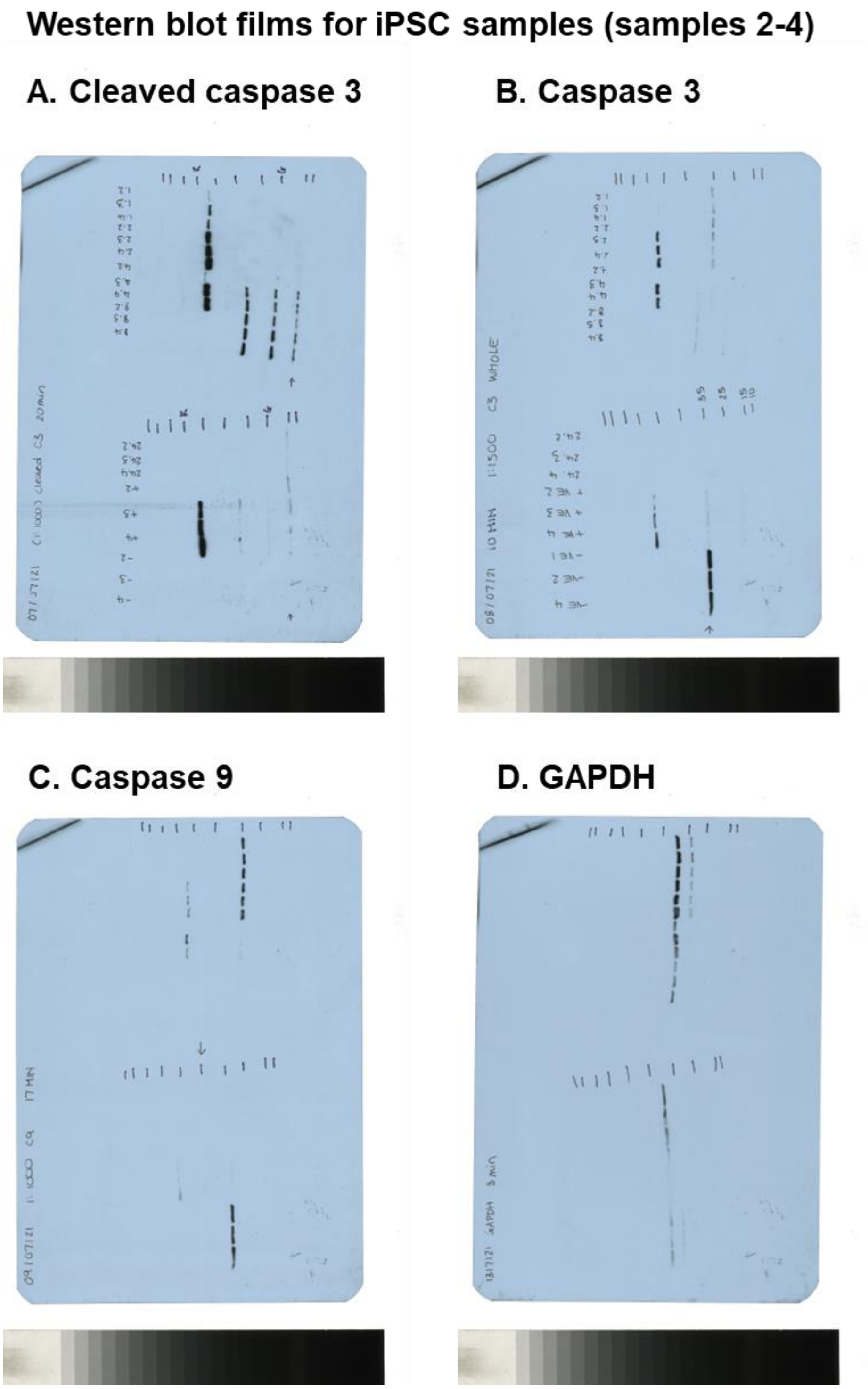

Figure S10: Raw western blot film scans used to assess anoikis activation in remaining iPSC samples. iPSC controls and suspension samples were characterised for the by the expression downstream markers of apoptosis, inactive caspases (A) 3 and (B) 9, (C) active cleaved caspase 3. (D-E) Protein quantification was normalised to the expression of housekeeper GAPDH. Negative control (-) refers to cells cultured on 2D tissue culture plastic surfaces, Positive control (+) for the onset of cell death were treated with 100 µM etoposide for 4 hours, Hours (h) refers to the amount of time samples were held in suspension to induce anoikis, Cleaved caspase 3 (cleaved C3).

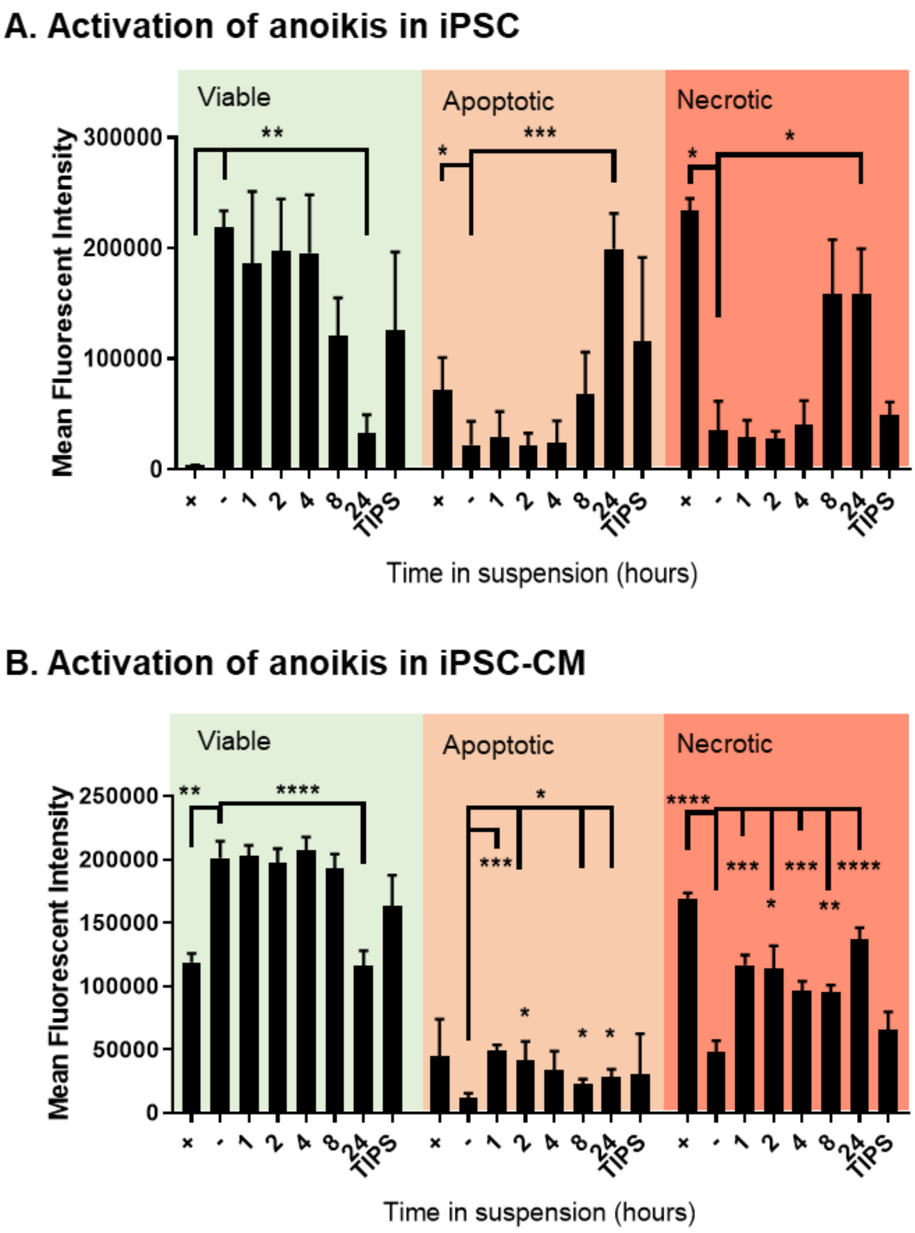

Figure S11: Flow cytometry assessment of anoikis of iPSC and iPSC- CM pre- and post-attachment to TIPS microspheres. (A) iPSC and (B) iPSC-CM were cultured in suspension up to 24 hours or attached to TIPS microspheres for 24 hours. iPSC and CM-iPSC attached onto 5 µg/ml VTN-N coated 6-well tissue culture plates were used as negative controls. Positive controls were treated with 10 µM staurosporine for 24 hours. Data are presented as mean ±SD. The significance of the data was calculated two-way ANOVA with Dunnett's post-hoc correction. (n=4-6, *P≤0.05, **P≤0.01, ***P≤0.001,****P≤0.0001, statistics to matched negative control).

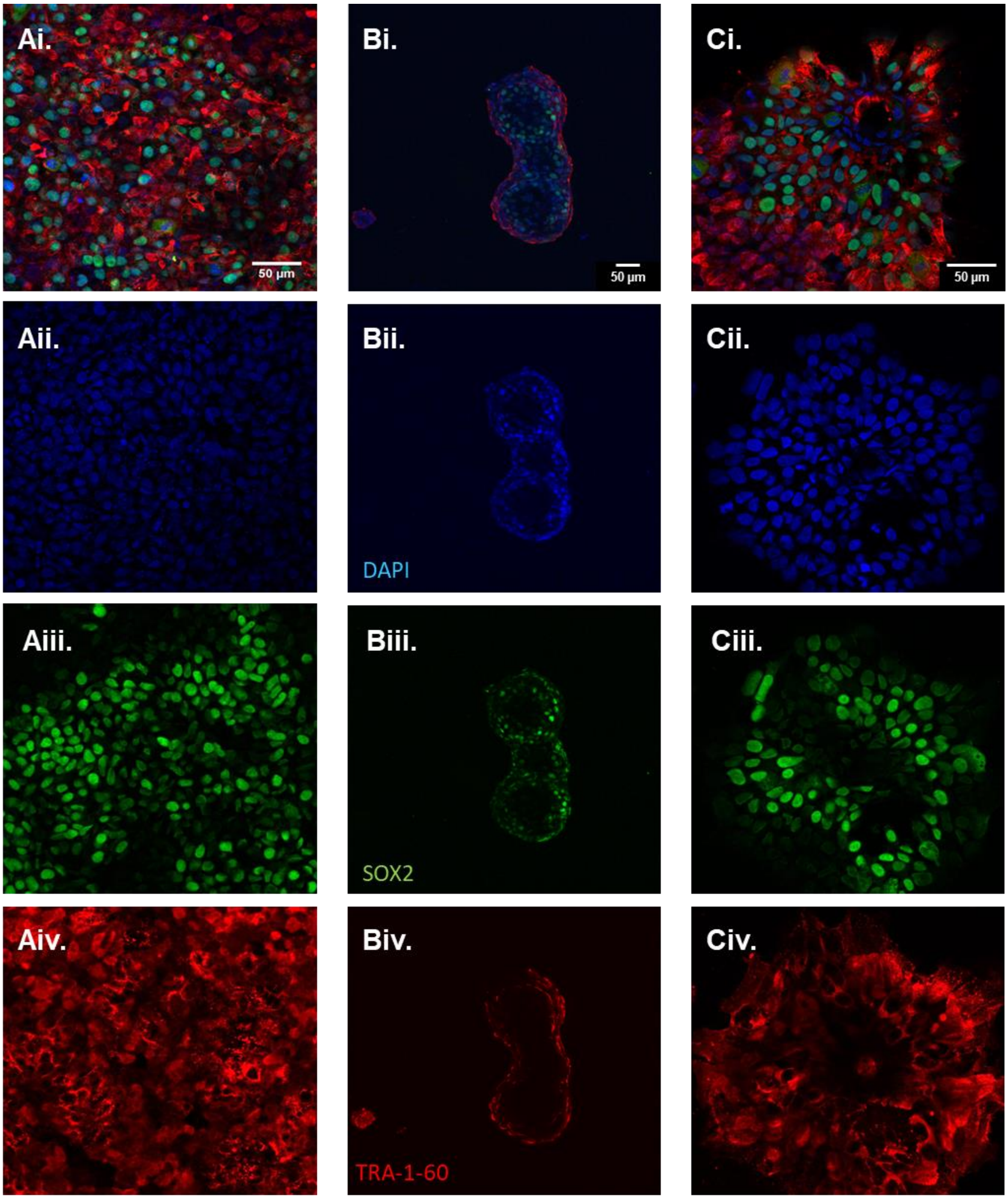

Figure S12: Immunofluorescence analysis of iPSC pluripotency. Retention of iPSC pluripotency markers, SOX2 (green) TRA-1-60 (red) and DAPI (blue), in (A) 2D control tissue culture plastic conditions, (B) attached to TIPS microcarriers and (C) migrated for 72 hours, off TIPS microcarriers and onto 2D control conditions. Scale bars represent 50 µm.

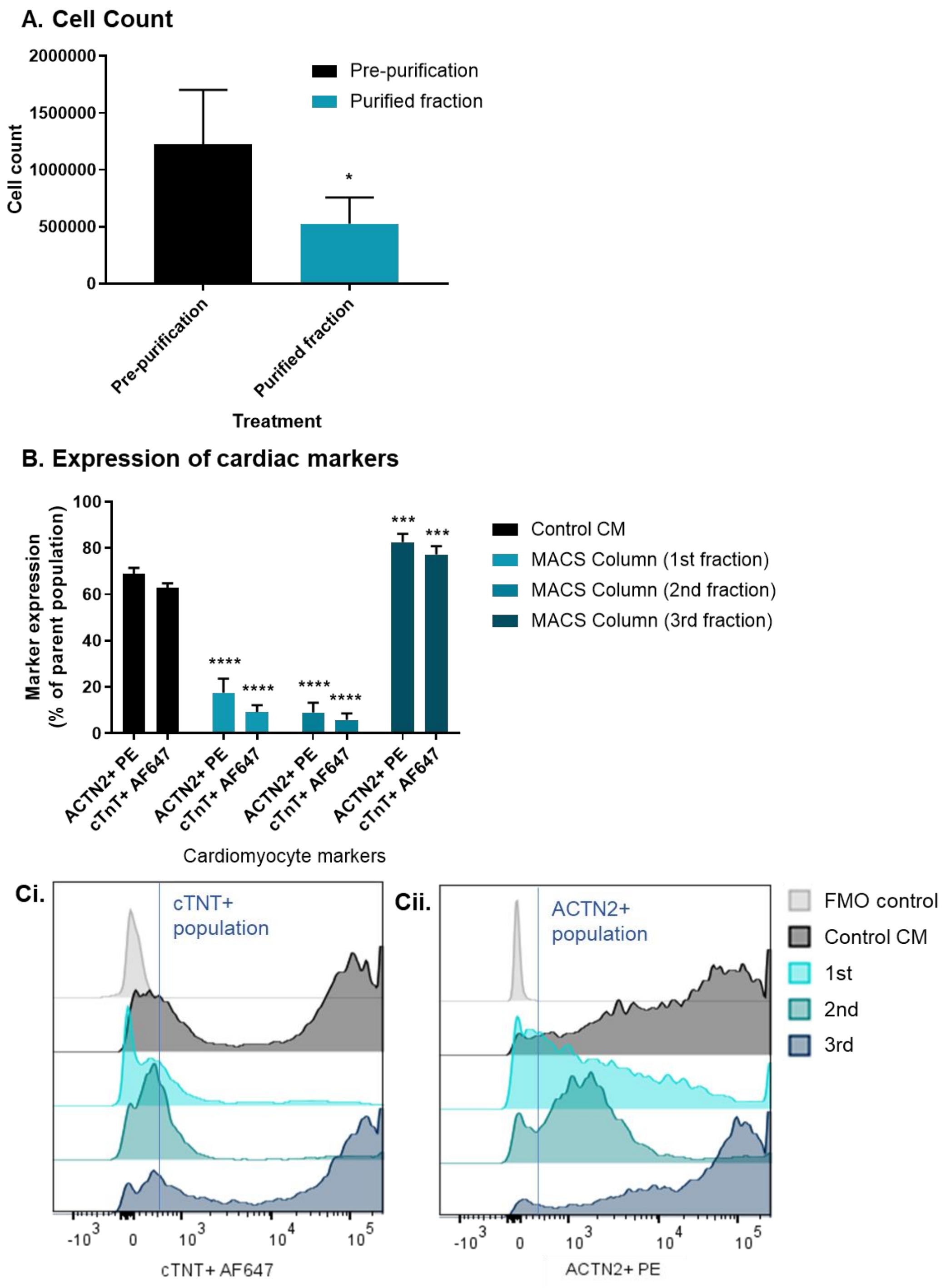

Figure S13: Characterisation of magnetically purified iPSC-CM. iPSC-CM were purified on day 18 of differentiation and marker expression was quantified immediately after purification. (A) Cell count pre- and post- enrichment. (B) Flow cytometric characterisation of the expression of cardiac markers (Ci) cTNT and (Cii) ACTN2 in each purification fraction. The non-CM were collected from the MACS column, as the 1st fraction. The second step positively selected CMs, flushing out the non-CM. The flushed out non-CM were collected as the 2nd fraction. Ultimately, the magnetically selected CM, and final product, were collect as the 3rd and final fraction. Data are presented as mean ±SD. The significance of the data was calculated (A) Unpaired Two-tailed t-test and (B) two-way ANOVA with Sidak's post-hoc correction. (n=4, *P≤0.05, ***P≤0.001, ****P≤0.0001, statistics to matched negative control).

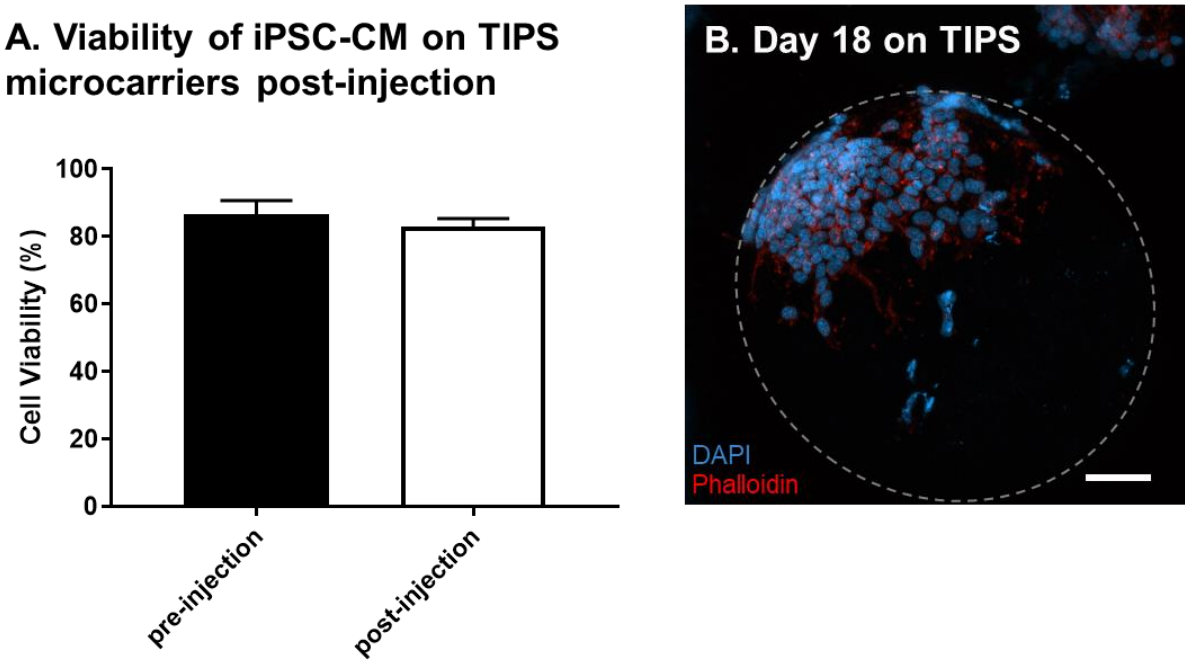

Figure S14: Further assessment of injectable iPSC-CM cellularised TIPS microcarriers. 1×10^6 enriched Day 18 iPSC-CM were seeded on 20 mg of microcarriers and incubated under static dynamic conditions for 24 hours. The media was replaced, and the cells were left to recover for another 72 hours. 4 days after cell seeding, the sample was resuspended in 600 µL of 60% GranuGel® and injected through a 23G needle capped syringe into a 24 well low-bind plate and the samples were immediately analysed for cell viability or cultured in EB 2% media for an additional 14 days. (A) Viability of iPSC-CM on TIPS microcarriers pre- and post-injection. Percentage cell viability in the sample was quantified using a Chemometec automated cell counter, with viability determined by the use of fluorescent dyes acridine orange and DAPI. (B) Confocal micrograph of iPSC-CM cultured on TIPS microcarriers for 18 days. TIPS microcarrier outlined by dotted grey line. The samples were fixed and stained for nuclear (DAPI blue) and cytoskeleton (phalloidin red) markers. Scale bar represents 50 µm. Data are presented as mean ±SD. n=2.

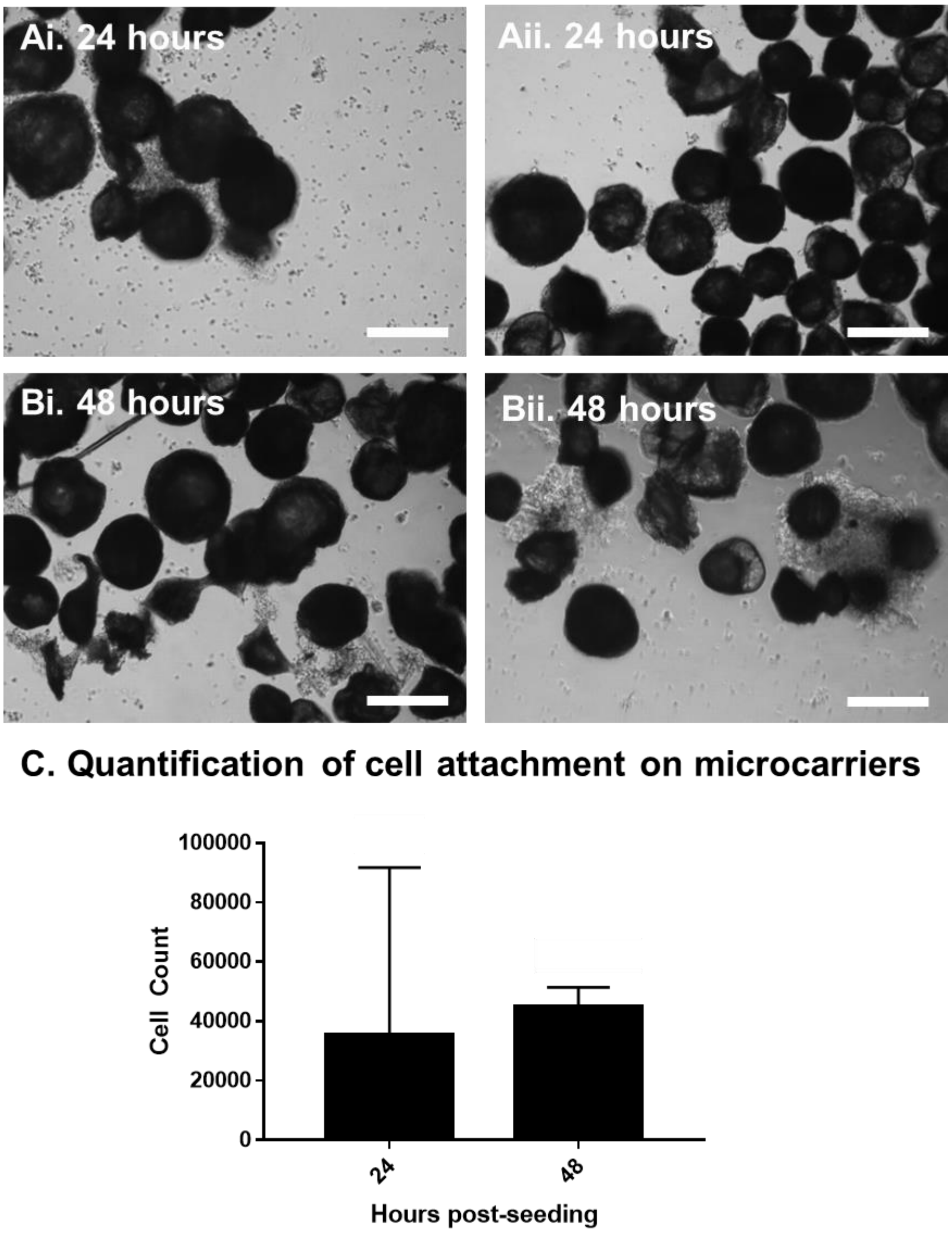

Figure S15: Late iPSC-CM do not expand on TIPS microcarriers. 2×10^5 day 18 iPSC-CM were seeded on 20 mg of <250 µm 2% 7507 TIPS microcarriers coated with 5µg/ml VTN-N and attached under static dynamic conditions. Light microscopy images show iPSC-CM attached to TIPS microcarriers after (A) 24 and (B) 48 hours post- seeding. Scale bars represent 100 µm. (C) Quantification of cell attachment suggests that the fraction of cells that attaches the microspheres, does not expand. Data are presented as mean ±SD. The significance of the data was calculated by two-tailed non-parametric t-test with Mann-Whitney post-hoc analysis. (n=3-4, P=0.34).
